# Time‐encoded ASL reveals lower cerebral blood flow in the early AD continuum

**DOI:** 10.1002/alz.14059

**Published:** 2024-07-03

**Authors:** Carles Falcon, Paula Montesinos, Lena Václavů, Michalis Kassinopoulos, Carolina Minguillon, Karine Fauria, Diego Cascales‐Lahoz, José Contador, Aida Fernández‐Lebrero, Irene Navalpotro, Albert Puig‐Pijoan, Oriol Grau‐Rivera, Gwendlyn Kollmorgen, Clara Quijano‐Rubio, José Luis Molinuevo, Henrik Zetterberg, Kaj Blennow, Marc Suárez‐Calvet, Matthias J. P. Van Osch, Javier Sanchez‐Gonzalez, Juan Domingo Gispert

**Affiliations:** ^1^ Barcelonaβeta Brain Research Center (BBRC) Pasqual Maragall Foundation Barcelona Spain; ^2^ Centro de Investigación Biomédica en Red de Bioingeniería Biomateriales y Nanomedicina (CIBER‐BBN) Instituto de Salud Carlos III Madrid Spain; ^3^ Neuroimagen de Enfermedades Neurodegenerativas y Envejecimiento Saludable Hospital del Mar Research Institute Barcelona Spain; ^4^ Philips Healthcare Iberia María de Portugal Madrid Spain; ^5^ Department of Radiology, C. J. Gorter MRI Center Leiden University Medical Center Leiden Netherlands; ^6^ Centro de Investigación Biomédica en Red de Fragilidad y Envejecimiento Saludable (CIBERFES) Instituto de Salud Carlos III Madrid Spain; ^7^ Servei de Neurologia Hospital del Mar Pg. Marítim de la Barceloneta Barcelona Spain; ^8^ Roche Diagnostics GmbH Penzberg Germany; ^9^ Roche Diagnostics International Ltd Rotkreuz Switzerland; ^10^ Clinical Neurochemistry Laboratory Sahlgrenska University Hospital Mölndal Sweden; ^11^ Department of Psychiatry and Neurochemistry Institute of Neuroscience and Physiology The Sahlgrenska Academy at University of Gothenburg Mölndal Sweden; ^12^ UK Dementia Research Institute at University College London (UCL) London UK; ^13^ Department of Neurodegenerative Disease UCL Institute of Neurology London UK; ^14^ Hong Kong Center for Neurodegenerative Diseases Clear Water Bay Hong Kong China; ^15^ Wisconsin Alzheimer's Disease Research Center University of Wisconsin School of Medicine and Public Health University of Wisconsin‐Madison Madison Wisconsin USA

**Keywords:** Alzheimer's disease, amyloid beta, magnetic resonance imaging, neurodegeneration, neurofilament light, p‐tau, single‐postlabel delay arterial spin labeling, synaptic dysfunction, tau proteins

## Abstract

**INTRODUCTION:**

Cerebral blood flow (CBF) is reduced in cognitively impaired (CI) Alzheimer's disease (AD) patients. We checked the sensitivity of time‐encoded arterial spin labeling (te‐ASL) in measuring CBF alterations in individuals with positive AD biomarkers and associations with relevant biomarkers in cognitively unimpaired (CU) individuals.

**METHODS:**

We compared te‐ASL with single‐postlabel delay (PLD) ASL in measuring CBF in 59 adults across the AD continuum, classified as CU amyloid beta (Aβ) negative (−), CU Aβ positive (+), and CI Aβ+. We sought associations of CBF with biomarkers of AD, cerebrovascular disease, synaptic dysfunction, neurodegeneration, and cognition in CU participants.

**RESULTS:**

te‐ASL was more sensitive at detecting CBF reduction in the CU Aβ+ and CI Aβ+ groups. In CU participants, lower CBF was associated with altered biomarkers of Aβ, tau, synaptic dysfunction, and neurodegeneration.

**DISCUSSION:**

CBF reduction occurs early in the AD continuum. te‐ASL is more sensitive than single‐PLD ASL at detecting CBF changes in AD.

**Highlights:**

- Lower CBF can be detected in CU subjects in the early AD continuum.
- te‐ASL is more sensitive than single‐PLD ASL at detecting CBF alterations in AD.
- CBF is linked to biomarkers of AD, synaptic dysfunction, and neurodegeneration.

## 1 INTRODUCTION

Alzheimer's disease (AD) is a progressive neurodegenerative condition and the most common cause of dementia.^1^ It is characterized by the presence of (1) amyloid beta (Aβ) pathology, which can be present in cognitively unimpaired (CU) individuals decades before the clinical symptoms appear; and (2) tau pathology, which closely precedes neurodegeneration, cognitive and functional decline.^2^ Aβ and tau pathology can be detected in vivo through fluid (cerebrospinal fluid [CSF] and blood) and imaging (positron emission tomography [PET]) biomarkers.^3^ In addition, biomarkers of synaptic dysfunction and neurodegeneration are altered in CU individuals with abnormal AD biomarkers.^4^, ^5^

Cerebral blood flow (CBF) is reduced in cognitively impaired (CI) AD patients^6^ in comparison with healthy controls, but the extent to which CBF is reduced in CU individuals with altered amyloid beta (Aβ+) biomarkers is controversial. One study^7^ reported lower CBF in early asymptomatic CU Aβ+ individuals in comparison with healthy CU Aβ− controls in the entorhinal cortex, an area of the brain associated with memory formation and early tau accumulation in AD.^8^, ^9^, ^10^ However, most studies have either failed to capture in CU individuals the association of CBF reduction with markers of AD, synaptic dysfunction, and neurodegeneration^11^, ^12^, ^13^, ^14^ or have only found CBF associations with the interaction of Aβ levels and cardiovascular risk factors.^15^

CBF can be measured using different techniques, such as PET, single photon emission computerized tomography (SPECT), and arterial spin labeling (ASL) magnetic resonance imaging (MRI).^16^ These three techniques are reliable and detect similar regions of CBF reduction in the parietal and temporal areas of the brains in CI AD patients^17^, ^18^; however, they have different costs and safety profiles. In particular, ASL MRI is more convenient than PET and SPECT because of its reduced cost, noninvasiveness, widespread availability, and lack of ionizing radiation and/or contrast medium injection.^19^

In 2015, the Perfusion Study Group of the International Society for Magnetic Resonance in Medicine (ISMRM) and the European Consortium for ASL in Dementia published their recommendation for the use of single postlabel delay (PLD) ASL^20^; however, this technique has limitations, the main one being its dependency on the need to select one, optimal PLD. To overcome this main limitation, multiple‐PLD ASL methods have been developed.^21^, ^22^ Among them, time‐encoded ASL (te‐ASL) is the most time‐efficient approach, with a lower sensitivity to potential tagged blood arrival differences, which allows for more accurate CBF estimates.^22^ te‐ASL can improve the estimation of CBF by accounting for between‐group, between‐subject, and intracerebral variability in the arterial transit time (ATT), that is, the time it takes for labeled blood to reach the brain regions where it will be read out. To date, there is little evidence of the role of te‐ASL in the field of AD research.^23^ We hypothesized that te‐ASL could be more sensitive than single‐PLD ASL at detecting CBF differences between CU Aβ− individuals and both CU Aβ+ CU individuals and CI Aβ+ AD patients and, thus, could help answer the question of whether CBF reduction occurred early in the AD continuum.

Therefore, in this study, we sought to (1) assess the sensitivity of te‐ASL in detecting CBF reductions in CI Aβ+ AD patients; (2) detect a possible CBF reduction in CU Aβ+ individuals; and (3) study CBF association with markers of AD, synaptic dysfunction, neurodegeneration, and cognition in all CU individuals. To address our first aim, we compared te‐ASL head to head with single‐PLD ASL in detecting CBF reductions in CI Aβ+ individuals (using CU Aβ− individuals as control). To address our second aim, we compared te‐ASL head to head with single‐PLD ASL in detecting CBF reductions, but this time in CU Aβ+ individuals (using CU Aβ− individuals as control). Finally, to meet our third aim, we investigated the association of CBF with markers of AD, cerebrovascular disease, synaptic dysfunction, neurodegeneration, and cognition in CU Aβ+ and CU Aβ− individuals.

## 2 METHODS

### 2.1 Study design and participants

This was an observational, cross‐sectional, case–control study that included 59 adults aged ≥60 years who were divided into three groups depending on (1) a clinical diagnosis of mild cognitive impairment (MCI patients) or AD dementia (CI patients) or the absence of it (CU participants); and (2) the presence of altered levels of CSF Aβ42/40 (≤0.071, as defined by Milà‐Alomà and colleagues,^4^ Aβ+) or the absence of it (Aβ−).

Of the 59 participants, 24 were healthy CU Aβ− controls, 18 were CU Aβ+ individuals, and 17 were CI Aβ+ patients. Eleven CU Aβ− participants belonged to the Alzheimer and Families (ALFA) ALFA cohort of the Barcelonaβeta Brain Research Center (BBRC) and had normal cognition and a lumbar puncture without abnormal levels of CSF Aβ42/40 in the 30 months before the start of the study.^24^ The remaining 13 participants were recruited from the BBRC registry of volunteers. All 18 CU Aβ+ participants belonged to the ALFA cohort of the BBRC.^24^ They had to present normal cognition and a lumbar puncture with abnormal levels of CSF Aβ42/40 in the 30 months before the start of this study. One of the 18 CU Aβ+ individuals did not have CSF biomarkers available but was added to this group on the basis of high levels of Aβ PET Centiloid (64.1 CL). For secondary analyses, we split the CU Aβ+ group into tau‐negative (A+T−) and tau‐positive (A+T+) according to pre‐established CSF pTau181 cut‐offs.^4^ All inclusion and exclusion criteria were previously described in our ALFA study.^24^

We recruited the 17 participants of the CI Aβ+ group from the Medical Research Unit at the Hospital del Mar in Barcelona. They had to be MCI or AD patients, as defined by the International Working Group 2 criteria.^25^ Moreover, they had to be positive for CSF AD biomarkers in the 30 months before the start of this study. Participants or their legal representatives signed a written consent form to take part in the study, which was previously approved by the Hospital del Mar Research Institute ethics committee. We conducted the study in compliance with the current version of the Declaration of Helsinki,^26^ the Ethical Guidelines for Epidemiological Studies,^27^ and applicable local legal and regulatory requirements for biomedical research.

To maintain an unbiased study design and data interpretation, we did not consider race, gender, or any other factors not listed in the inclusion or exclusion criteria for patient selection.

**RESEARCH IN CONTEXT**

1. **Systematic review**: We reviewed the literature on the changes in cerebral blood flow (CBF) in Alzheimer's disease (AD). There are few inconsistent reports of CBF reductions in cognitively unimpaired (CU) amyloid‐positive individuals. Furthermore, no studies used time‐encoded arterial spin labeling (te‐ASL) to measure CBF across the AD *continuum*.
2. **Interpretation**: CBF reduction occurs earlier in the AD *continuum* than previously thought. On the contrary to single‐postlabel delay (PLD) ASL, te‐ASL is able to detect CBF reductions in early asymptomatic stages of AD. CBF reduction is associated with AD, synaptic dysfunction, and neurodegeneration biomarkers in CU participants.
3. **Future directions**: CBF measured using te‐ASL is a promising biomarker of AD progression. Further large‐scale longitudinal studies are needed to strengthen our findings.

### 2.2 MRI acquisition and processing

#### 2.2.1 Structural MRI acquisition

We used structural MRI to measure neurodegeneration markers, such as hippocampal volume and the AD signature (which is based on the cortical thickness of AD‐vulnerable brain regions^28^), and white matter hyperintensities (WMH) as a marker of cerebrovascular disease. Data were acquired using a 3T Philips Ingenia CX scanner at the neuroimaging unit at BBRC using a 32‐channel head coil. High‐resolution structural images were acquired using a 3D T1‐weighted (T1w) Turbo Field Echo pulse sequence (repetition time (TR) / echo time (TE) / inversion time (TI) = 9.9/4.6/900 ms, flip angle = 8°, field of view (FOV) = 180 × 240 × 240 mm^3^, sagittal orientation, voxel size = 0.75 × 0.75 × 0.75 mm^3^). Fluid‐attenuated inversion recovery (FLAIR) images were acquired using a 3D flip angle sweep turbo spine echo pulse sequence (TR/TE/TI = 5000/312/1700 ms, flip angle = 90°, FOV = 180 × 250 × 250 mm^3^, sagittal orientation, voxel size = 1.0 × 0.98 × 0.98 mm^3^). T2w images were acquired using a turbo spin echo pulse sequence (TR/TE = 2500/264 ms, flip angle = 90°, FOV = 180 × 250 × 250 mm^3^, sagittal orientation, voxel size = 0.5 × 0.98 × 0.98 mm^3^). Additional T1w images were acquired in the session of the ASL MRI acquisition to facilitate the registration of ASL perfusion maps to the Montreal Neurological Institute (MNI) space, using the following magnetic resonance (MR)‐ parameters: TR/TE/TI = 9.8/3.0/900 ms, flip angle = 8°, FOV = 180 × 240 × 240 mm^3^, sagittal orientation, voxel size = 1.2 × 0.68 × 0.68 mm^3^.

#### 2.2.2 ASL MRI acquisition

We used ASL MRI to measure CBF. Participants were instructed to rest with their eyes open and not to sleep or think about anything in particular during the acquisition. We performed perfusion MRI using a time‐encoded pseudo‐continuous ASL sequence (te‐ASL) with background suppression and the following acquisition parameters: TR = 4600 ms, TE = 12 ms, flip angle = 90°, FOV = 220 × 220 × 120 mm^3^, voxel size = 2.29 × 2.29 × 6.0 mm^3^, 20 slices in ascending order, thickness/gap = 6 mm/0 mm, label duration = 3600 ms, acquired with seven different PLD times (200, 350, 500, 700, 1000, 1400, and 2000 ms) and label durations (150, 150, 200, 300, 400, 600, and 1800 ms), time‐encoded factor = 8, Hadamard‐8 labeling scheme, scan duration = 5 min 31 s. We also acquired a magnetization equilibrium (M0) 2D image using the same parameters as te‐ASL, except that TR/TE was 2000/10 ms, and labeling and background suppression were switched off (scan duration = 14 s).

#### 2.2.3 Postprocessing of te‐ASL MRI

We used T1w images for tissue segmentation and for facilitating the registration of the ASL perfusion maps to the MNI space. We decoded the te‐ASL raw images into ASL‐signal images using an in‐house script in MATLAB and obtained CBF and ATT maps from the ASL‐signal, T1w, and M0 images via the Bayesian Inference for Arterial Spin Labeling (BASIL) toolbox distributed within the FSL package (https://fsl.fmrib.ox.ac.uk/fsl/fslwiki/BASIL).^29^ Then we normalized the CBF and ATT maps in structural space to MNI space using the flow fields resulting from the standard DARTEL normalization of T1 images to MNI space, as implemented in Statistical Parametric Mapping software (SPM12; https://www.fil.ion.ucl.ac.uk/spm/). Finally, we smoothed these MNI normalized maps with a Gaussian kernel of 12 mm full width at half maximum (FWHM). To focus the analysis on regional differences instead of global differences, we normalized the CBF intensity values to the mean value in the cerebellar gray matter, as previously proposed.^11^

#### 2.2.4 Postprocessing of single‐PLD ASL MRI

To compare the performance of te‐ASL (which relies on multiple subboluses with different PLDs) to single‐PLD ASL, we estimated an additional set of CBF maps from te‐ASL data considering only the longest PLD of the te‐ASL schema (perfusion block, nominal PLD time of 2000 ms, labeling duration of 1800 ms), in agreement with the 2015 recommendations.^20^ Specifically, we created single‐PLD CBF maps on the basis of the difference of label and control images of the raw te‐ASL images corresponding to the last PLD. As we did for te‐ASL, we normalized CBF maps from single‐PLD to MNI space using the flow fields generated with DARTEL and spatially smoothed them with a Gaussian kernel of 12 mm FWHM. Again, we normalized CBF intensity values to the mean CBF value in the cerebellar gray matter.^11^

### 2.3 MRI quantifications

#### 2.3.1 Quantifications from structural MRI

##### Hippocampal volume

We segmented the hippocampus on T1w MRI images using FreeSurfer version 7.0 twice: once with T1w images from the structural MRI session and once with the T1w images from the ASL MRI session. A bilateral hippocampal volume variable was constructed by summing up the volumes in the left and right hemispheres. We then employed linear regression analysis to remove the effect of total intracranial volume (TIV), which is a measurement derived from FreeSurfer as well, and construct the TIV‐adjusted hippocampal volume measurement. TIV‐adjusted hippocampal volumes reflect the deviation in a participant's hippocampal volume from what is expected from their TIV.^30^

##### AD signature

We computed Jack and colleagues’ (2017)^28^ AD‐signature composite using FreeSurfer version 7.0 twice: once with T1w images from the structural MRI session and once with the T1w images from the ASL MRI session of the current study. This measure is based on the surface‐area‐weighted average of the mean cortical thickness in the following regions of interest: entorhinal, inferior temporal, and middle temporal cortices and the fusiform gyri.^31^

##### White matter hyperintensities

We derived WMH segmentation images on the basis of T1w, T2w, and FLAIR MRI images using the Bayesian Model Selection method.^29^ Then we derived total WMH volumes by multiplying the total number of voxels labeled as white matter lesions by voxel dimensions. Finally, we divided total WMH volumes by TIV to account for interindividual variability in TIV and obtained volume percentages of WMH.

#### 2.3.2 Region‐specific CBF measurements from single‐PLD ASL and te‐ASL MRI

CBF measurements from single‐PLD ASL and te‐ASL were averaged within an a priori mask that included areas of the brain associated with CBF reduction in AD, as previously reported.^11^ The a priori mask consisted of the following parcels of the Harvard–Oxford atlas^32^: lateral and medial parietal cortex (anterior/posterior supramarginal gyri, angular gyrus, posterior cingulate, precuneus); lateral temporal cortex (anterior/posterior divisions of the middle and inferior temporal gyri); medial temporal cortex; hippocampus; and anterior/posterior divisions of the parahippocampal gyrus.

In addition to the a priori mask, two data‐driven masks were subsequently used to quantify mean CBF on the basis of te‐ASL data in all CU individuals. The first data‐driven mask consisted of areas where te‐ASL revealed significantly reduced CBF in CI Aβ+ individuals, in comparison with CU Aβ− individuals. The second data‐driven mask consisted of areas where te‐ASL revealed significantly reduced CBF in CU Aβ+ individuals in comparison with CU Aβ− individuals.

### 2.4 CSF biomarkers

In the two CU groups, CU Aβ− and CU Aβ+, we analyzed CSF biomarkers of AD (Aβ and phosphorylated tau proteins), synaptic dysfunction (growth‐associated protein 43 [GAP43], neurogranin, synaptosomal‐association protein 25 [SNAP25], and synaptotagmin‐1), and neurodegeneration (neurofilament light [NfL]), which were measured in previous studies.^4^, ^33^

Participants underwent lumbar puncture for CSF on average 18 ± 7 months before the ASL MRI data. Measurements were performed at the Clinical Neurochemistry Laboratory, Sahlgrenska University Hospital, Mölndal, Sweden. We measured core prototype CSF biomarkers using the NeuroToolKit (Aβ42, Aβ40, and NfL) and Elecsys (pTau181) on an automated cobas e 601 analyzer (Roche Diagnostics International Ltd, Rotkreuz, Switzerland). Aβ positivity was defined using pre‐established cut‐offs^4^ as CSF Aβ42/40 ≤ 0.071, and for secondary analyses, tau positivity was considered as CSF pTau181 > 24 pg/mL. Apart from CSF pTau181, we also measured CSF pTau217, pTau 231, and pTau235 since they were recently reported to increase in the early AD continuum.^34^ We measured CSF pTau217 with an in‐house Simoa assay, CSF pTau 231 with an ADx immunoassay,^34^ and CSF pTau235 with an in‐house Simoa assay, on an HD‐X instrument (Quanterix).^35^ For synaptic dysfunction, we measured GAP43 through ELISA, as described by Sandelius et al. in 2019,^36^ and SNAP25 and synaptotagmin‐1 by immunoprecipitation mass spectrometry, as described by Tible et al. in 2020.^37^

With respect to the CI Aβ+ participants recruited from the Hospital del Mar, we were provided with the tau positivity of each participant, which was based on the levels of pTau181 (CSF pTau181 > 69.9 pg/mL). pTau181 was measured using a Lumipulse G600II analyzer (Fujirebio, Belgium) as described by Puig–Pijoan et al. in 2002.^38^ Although several other CSF biomarkers were available for this group, they were not considered in the current study as they were derived using different procedures and were not easily comparable to those available for the CU participants.

### 2.5 Aβ PET

Aβ PET scans were acquired from CU individuals using [^18^F] flutemetamol as radiotracer after a cranial computed tomography scan for attenuation correction on a Biograph mCT scanner (Siemens Healthcare, Erlangen, Germany) at the Hospital Clínic, Barcelona, Spain. Aβ PET scans were acquired on average 15 ± 7 months before the ASL MRI data. Images were acquired for 20 min (four frames of 5 min each) 90 min after injection (mean ± SD 90.15 ± 7.36 min) of an IV bolus [^18^F] flutemetamol dose of 185 MBq (range 104.25 to 218.3 MBq, mean ± SD 191.75 ± 14.04 MBq). Processing was performed following a validated Centiloid pipeline^39^ with SPM12.^40^ Finally, we calculated Centiloid values from the mean values of the standard global cortical average target region and the whole cerebellum as reference region using the previously calibrated transformation.^39^

### 2.6 Cognition

Cognition was evaluated in CU individuals through a comprehensive neuropsychological assessment battery covering the following five cognitive domains^41^, ^42^, ^43^, ^44^, ^45^: attention (Wechsler Adult Intelligence Scale [WAIS]‐IV, Digit Span; Wechsler Memory Scale–Fourth Edition [WMS‐IV], Symbol Span; Trail Making Test [TMT]‐A); episodic memory (Free and Cued Selective Reminding Test, Memory Binding Test, WMS‐IV Logical Memory, NIH Toolbox Picture Sequence Memory Test); executive functioning (TMT‐B, WAIS‐IV Coding, WAIS‐IV Matrix Reasoning, and NIH Toolbox Flanker Inhibition Test); language (Semantic Fluency Test); and visual processing (WAIS‐IV Visual Puzzles, RBANS Judgement of Line Orientation).

We performed a cognitive evaluation on average 19 ± 6 months before obtaining the ASL MRI data and standardized the raw scores of each individual into a *z*‐score using the means and standard deviations obtained from a biomarker‐negative sample from our previous ALFA project cohort (*n* = 248) as a reference.^46^ In addition to the composite score of each cognitive domain, we created a global composite, termed the Preclinical Alzheimer Cognitive Composite (PACC), that summarizes cognitive status using variables that are especially sensitive for early AD.^47^ Further, the Mini–Mental State Examination (MMSE)^48^ was used to evaluate cognition in both CU^24^ and CI^38^ individuals.

### 2.7 *APOE* genotyping

In CU individuals, total DNA was obtained from cellular blood fraction by proteinase K digestion followed by alcohol precipitation. Samples were genotyped for two single‐nucleotide polymorphisms, rs429358 and rs7412, to define the apolipoprotein E (*APOE)*‐ε2, ε3, and ε4 alleles. *APOE* genotyping was performed in a similar manner for CI Aβ+ participants as well.^38^ In this study, participants were classified as *APOE*‐ε4 carriers (with one or two alleles) or ε4 non‐carriers.

### 2.8 Statistical analysis

We assessed demographic differences among study groups through analysis of variance (ANOVA) models and post hoc pairwise contrasts (or the non‐parametric equivalent when appropriate) for continuous variables and through chi‐squared tests for categorical variables.

We compared mean CBF over the a priori mask among study groups with one way three‐group (CU Aβ−, CU Aβ+, and CI Aβ+) ANOVA and post hoc pairwise comparisons, using sex and age as confounders. The statistical significance threshold was set to *p* < 0.05.

For the ASL images, we conducted a voxel‐wise analysis of spatially normalized CBF maps with SPM12 (www.fil.ion.ucl.ac.uk/spm). To this end, groups were entered as categorical variables in the general linear model in SPM12, and age and sex were included as independent regressors. We applied post hoc contrasts to render between‐group differences. We set the statistical threshold of significance to *p *< 0.001 uncorrected for multiple comparisons considering a minimum cluster size of 100 voxels (0.33 cm^3^ approximately).^49^ In a similar manner, we investigated the effect of age and sex on the ATT maps obtained with te‐ASL. Additionally, we examined between‐group differences in the ATT maps, including age and sex as independent regressors.

For the biomarker analyses, outliers, defined as values of a variable deviating more than three standard deviations from the mean, were excluded before conducting a given test.

Finally, we sought associations of individual CBF means in the brain regions inside the a priori and data‐driven masks with biomarkers of AD, synaptic dysfunction, neurodegeneration, vascular pathology, and cognition through Pearson correlation with a significance level of *p *< 0.05, after regressing out age and sex effects. *p*‐values were adjusted for multiple comparisons using the Benjamini–Hochberg method.

## 3 RESULTS

### 3.1 Participant demographics and characteristics

Table 1 describes the participant demographics (age and sex) for the three groups and other disease characteristics for the two CU groups. There are limited data related to disease characteristics for the CI group as these participants were recruited from another study at Hospital del Mar and only underwent ASL MRI acquisition for the purposes of this study. There was a significant difference in age between the CI Αβ+ group and the other two groups (*p *= 0.004 for CU Αβ− and *p *< 0.001 for CU Αβ+). Also, the percentage of women was significantly higher in the CU Aβ+ group (83.3%) than in the CI Aβ+ group (47.1%) (*p *= 0.024) (Table 1). Moreover, 52.9% of the CU Aβ+ and 94.1% of the CI Aβ+ individuals were positive for pTau181, whereas none in the CU Aβ− group was (*p* ≤ 0.003). Also, we found significantly more carriers of *APOE‐*ε4 (a well‐known risk factor for AD^50^, ^51^, ^52^) in the CU Αβ+ (66.7%) than in the CU Αβ− group (27.3%) (*p *= 0.039). No differences were observed in TIV‐adjusted hippocampal volume, AD signature, WMH, PACC, and MMSE score between the CU Αβ− and the CU Αβ+ groups (Table 1). As expected, CI Αβ+ individuals exhibited significantly lower TIV‐adjusted hippocampal volume, AD signature, and MMSE scores than CU individuals (*p* < 0.001).

**TABLE 1:** Study participants’ demographics and characteristics of Alzheimer's disease.

	CU Aβ− (*n* = 24)	CU Aβ+ (*n* = 18)	CΙ Aβ+ (*n* = 17)	CU Aβ− versus CU Aβ+ (*p*‐value)	CU Aβ− versus CI Aβ+ (*p*‐value)	CU Aβ+ versus CI Aβ+ (*p*‐value)
Age, years	67.4 (4.2) [61, 75]	66.1 (2.9) [60, 71]	73.1 (6.5) [61, 85]	0.257	0.004	<0.001
Females, *N* (%)	16 (66.7)	15 (83.3)	8 (47.1)	0.224	0.209	0.024
CSF Aβ42/40	0.088 (0.009)	0.041 (0.010)	N/A	<0.001	N/A	N/A
Aβ PET (CL)	−4.7 (8.4)	40.9 (18.3)	N/A	<0.001	N/A	N/A
Individuals positive for CSF pTau181, *N* (%)	0/11 (0.0%)	9/17 (52.9%)	16/17 (94.1%)	0.003	<0.001	<0.001
*APOE*‐ε4 carriers, N (%)	3/11 (27.3%)	12/18 (66.7%)	10/17 (58.8%)	0.039	0.180	0.407
TIV‐adjusted hippocampal volume (structural MRI session), mm^3^	7284.1 (714.8)	7318.3 (552.3)	N/A	0.888	N/A	N/A
TIV‐adjusted hippocampal volume (ASL MRI session), mm^3^	7250.1 (528.3)	7139.4 (669.7	5946.1 (910.6)	0.567	<0.001	<0.001
Volume percentage of WMH, %	0.2 (0.1)	0.3 (0.3)	N/A	0.113	N/A	N/A
AD signature (structural MRI session), mm	2.5 (0.1)	2.5 (0.1)	N/A	0.942	N/A	N/A
AD signature (ASL MRI session), mm	2.6 (0.1)	2.6 (0.1)	2.4 (0.2)	0.497	<0.001	<0.001
PACC (*z*‐score)	0.0 (0.7)	−0.5 (0.6)	N/A	0.079	N/A	N/A
MMSE score	29.3 (1.1) [27, 30]	28.8 (1.2) [27, 30]	21.5 (3.1) [17, 29]	0.333	<0.001	<0.001

*Note*: Data are expressed as mean (SD) for all measurements, and number/total available (percentage) for the variables “females,” “individuals positive for CSF pTau181,” and “*APOE*‐ε4 carriers.” The ranges for age and MMSE score are indicated in square brackets. The *p*‐values were computed with a *t* test for all the measurements and χ^2^ test for the variables “females,” “individuals positive for CSF pTau181,” and “*APOE*‐ε4 carriers.” CSF Aβ42/40 and CSF pTau181 were available for 11 out of 24 CU Aβ−, 17 out of 18 CU Aβ+, and all 17 CI Aβ+ participants; Aβ PET Centiloids for 10 CU Aβ− and 14 CU Aβ+ participants; *APOE*‐ε4 carrier status for 11 CU Aβ− and all CU (18) and CI (17) Aβ+ participants; TIV‐adjusted hippocampal volume (structural MRI session) for 13 CU Aβ− and 17 CU Aβ+ participants; TIV‐adjusted hippocampal volume (ASL MRI session) for all 59 participants; volume percentage of WMH for 13 CU Aβ− and 17 CU Aβ+ participants; AD signature (structural MRI session) for 13 CU Aβ− and 17 CU Aβ+ participants; AD signature (ASL MRI session) for all 59 participants; PACC for 11 CU Aβ− and 18 CU Aβ+ participants; and MMSE score for 11 CU Aβ−, 18 CU Aβ+, and 17 CI Aβ+ participants. Different CSF pTau181 cut‐offs were considered for tau positivity in CU and CI individuals (>24 pg/mL in CU individuals and >69.9 pg/mL in CI individuals), due to different protocols employed for the quantification of pTau181 levels.

Limited data are available for the CI Aβ+ group because these patients were recruited from another study at Hospital del Mar that used different protocols for quantifying CSF biomarkers.

Abbreviations: Aβ, amyloid beta; Aβ−, normal levels of Aβ proteins; Aβ+, altered levels of Aβ proteins; AD, Alzheimer's disease; *APOE*‐ε4, apolipoprotein E epsilon 4; CI, cognitively impaired; CSF, cerebrospinal fluid; CU, cognitively unimpaired; MMSE, Mini‐Mental State Examination; PACC, preclinical Alzheimer cognitive composite; TIV, total intracranial volume; WMH, white matter hyperintensities.

Finally, the CU Aβ+ group also presented significantly abnormal levels of all CSF biomarkers of AD, synaptic dysfunction, neurodegeneration, and cognition, in comparison with the CU Αβ− group (Table S1).

### 3.2 Effect of ATT

We examined group differences (CU Aβ+ vs CU Aβ−, CI Aβ+ vs CU Aβ−) on the mean ATT averaged within the a priori mask (areas of the brain previously associated with CBF reduction in AD) and found no significant differences (Figure S1). In addition, we looked for between‐group differences in the ATT maps, and no regional differences could be detected either.

To better understand the extent of variability in ATT across individuals, we examined the effect of age and sex on voxel‐wise ATT maps within the entire cohort. The association between age and ATT was found to be positive in various regions, including the anterior cingulate, medial frontal gyrus, precuneus, and lingual gyrus, as well as in the bilateral caudate, putamen, and thalamus (Figure S2). Furthermore, males exhibited higher ATT than females in multiple gray matter regions, encompassing the middle frontal gyrus, anterior cingulate, posterior insula, precuneus, inferior parietal lobe, angular gyrus, and middle temporal gyrus.

### 3.3 te‐ASL sensitivity in detecting lower CBF in CI Aβ+

**FIGURE 1:**
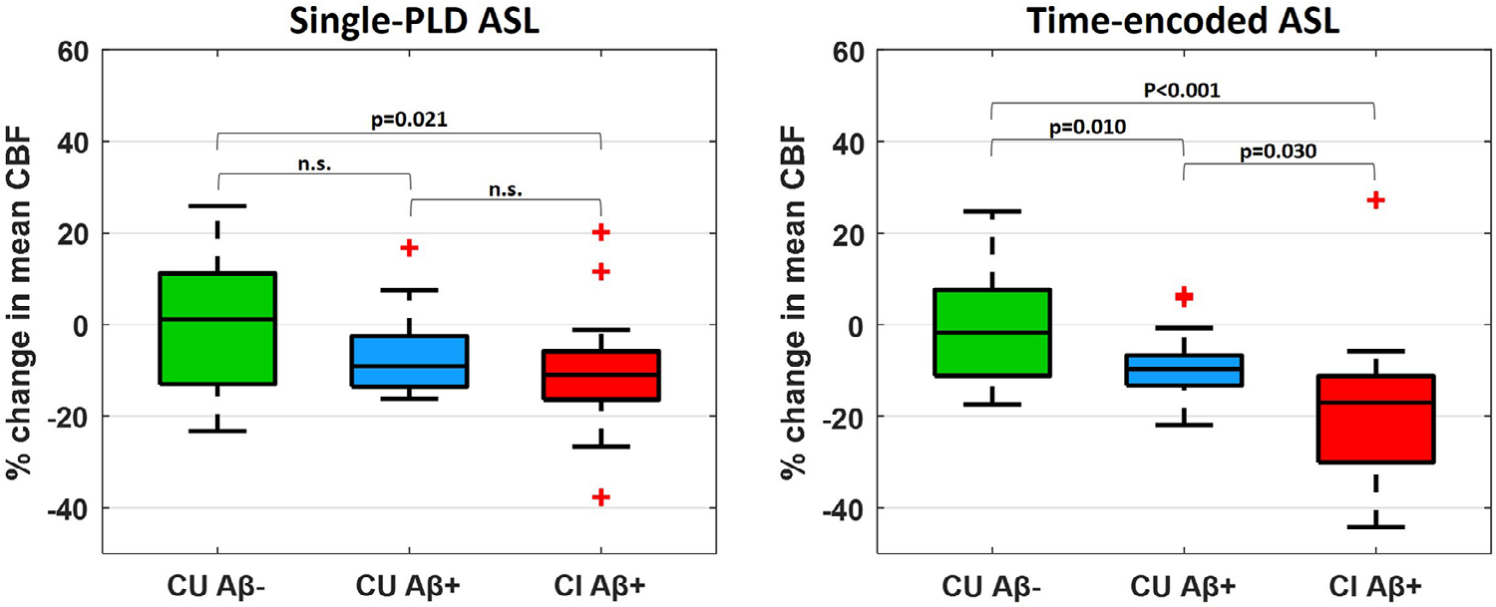
Percentage change in mean CBF in comparison with CU Aβ− group computed within an a priori mask using single‐PLD ASL and te‐ASL. The a priori mask consisted of areas of the brain associated with CBF reduction in AD, as previously reported.^11^ CBF values were normalized to those in the cerebellum to account for variability across individuals and are presented in percentage change with respect to the CU Aβ− group. The mean CBF quantified through single‐PLD ASL data was significantly lower in the CI Aβ+ (*n* = 17) group than in the CU Aβ− (*n* = 24) group. The mean CBF quantified through te‐ASL was significantly lower in the CI Aβ+ group than the CU Aβ+ (*n* = 18) and CU Aβ− groups; and it also was significantly lower in the CU Aβ+ group than in the CU Aβ− group. Red crosses above or below boxplots indicate outliers. Aβ, amyloid beta; Aβ−, normal levels of Aβ proteins; Aβ+, altered levels of Aβ proteins; AD, Alzheimer's disease; ASL, arterial spin labeling; CBF, cerebral blood flow; CI, cognitively impaired; CU, cognitively unimpaired; n.s., non‐significant; PLD, postlabel delay; te, time‐encoded.

To assess the sensitivity of te‐ASL in detecting CBF reductions in symptomatic AD, we compared te‐ASL head to head with single‐PLD ASL at detecting CBF reductions in CI Aβ+ individuals (using the CU Aβ− individuals as control). Specifically, we measured the mean percentage reduction in CBF inside the a priori mask in the CI Aβ+ group, in comparison with the CU Aβ− group. We found a reduction in CBF of 10.24% (95% CI [1.66%, 18.82%], *p *= 0.021) using single‐PLD ASL (Figure 1), although this finding did not survive multiple‐comparisons correction (*p*_FDR_ = 0.062). Conversely, te‐ASL presented a significant reduction of 18.03% (95% CI [8.66%, 27.40%], *p *< 0.001) even after multiple‐comparisons correction (*p*_FDR_ = 0.001). The reduced CBF values in CI Aβ+ could also be observed in subject‐level CBF maps using te‐ASL and, to a lesser extent, with single‐PLD ASL (Figure S3).

### 3.4 Lower CBF in CU Aβ+

**FIGURE 2:**
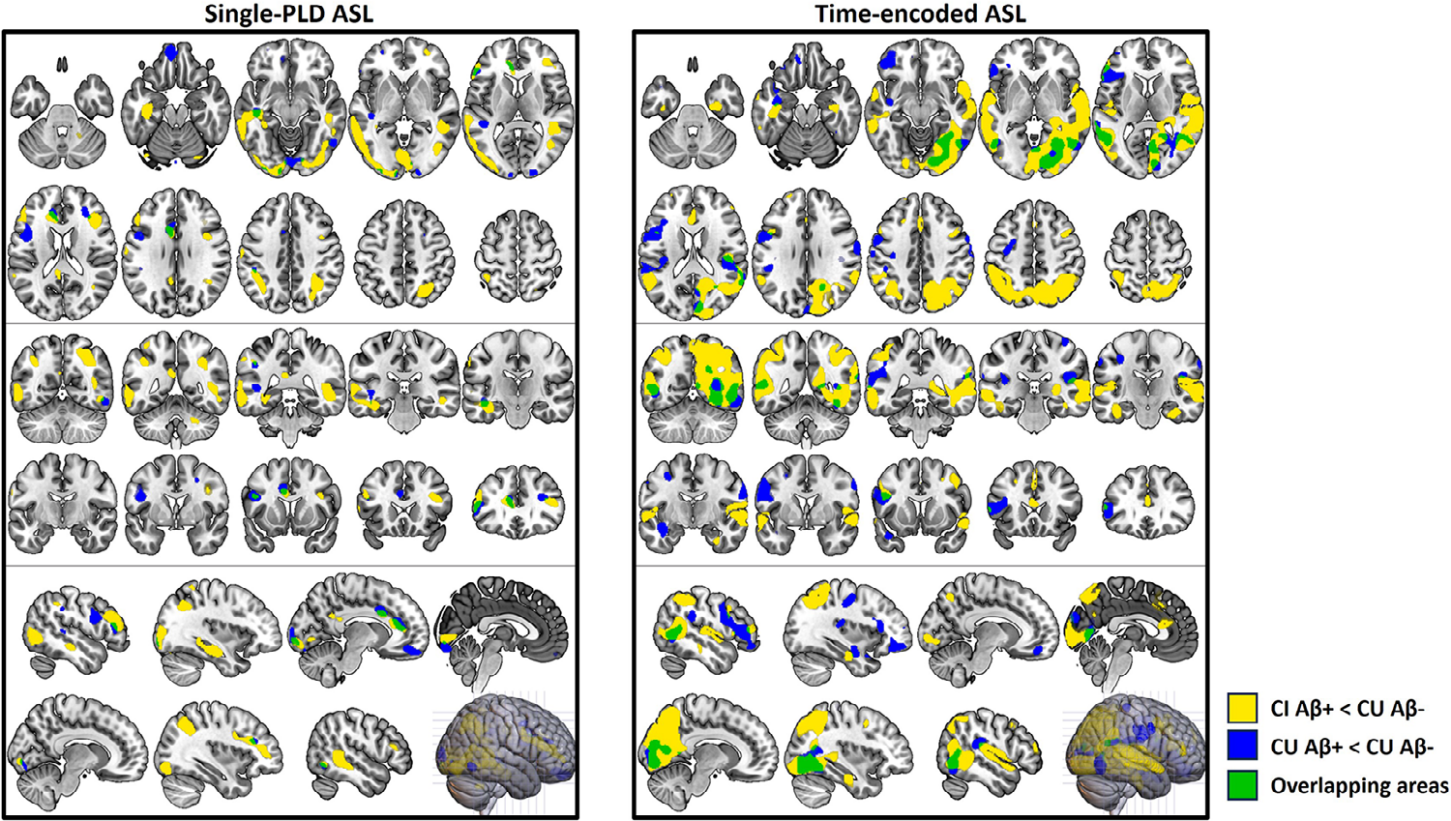
Areas of significant CBF reduction in CI Aβ+ and CU Aβ+ groups in comparison with CU Aβ− group using single‐PLD ASL and te‐ASL. We used a two‐sample *t* test with a statistical threshold of *p *< 0.001 uncorrected for multiple comparisons and removed clusters smaller than 100 voxels (∼0.33 cm^3^). The brain images are presented in the axial (upper two rows), coronal (middle two rows), and sagittal (lower two rows) views. The yellow color shows areas with reduced CBF in the CI Aβ+ in comparison to the CU Aβ− group, the blue color shows areas with reduced CBF in the CU Aβ+ in comparison to the CU Aβ− group, and the green color shows areas with reduced CBF in both CI Aβ+ and CU Aβ+ in comparison to the CU Aβ− group. Using te‐ASL, the CI Aβ+ and CU Aβ+ groups presented overlapping areas of CBF reduction in the bilateral posterior insula, cuneus, superior temporal gyrus, and right fusiform gyrus. The CI Aβ+ group also exhibited CBF reduction in lateral and medial regions of the temporal and parietal cortex, posterior cingulate, hippocampus, and divisions of parahippocampal gyrus, which are part of the areas of the brain associated with CBF reduction in AD, as previously reported.^11^ Neither of the two Aβ+ groups presented any regional increases in CBF as compared to the CU Aβ− group. The unthresholded statistical maps generated in this study are available at https://neurovault.org/collections/SAHGRKNE/. Aβ, amyloid beta; Aβ−, normal levels of Aβ proteins; Aβ+, altered levels of Aβ proteins; ASL, arterial spin labeling; CBF, cerebral blood flow; CI, cognitively impaired; CU, cognitively unimpaired; PLD, postlabel delay; te, time‐encoded.

To detect a possible CBF reduction in asymptomatic AD, we again compared te‐ASL head‐to‐head with single‐PLD ASL at detecting CBF reductions this time in CU Aβ+ individuals (using the CU Aβ− individuals as control) inside the a priori mask. The mean percentage reduction in CBF in the CU Aβ+ group was 6.87% (95% CI [−0.02%, 13.75%], non‐significant) using single‐PLD ASL, and 8.55% (95% CI [2.14%, 14.91%], *p *= 0.010) using te‐ASL (Figure 1). Finally, te‐ASL revealed overlapping brain areas where CBF reduction occurred in the CI Aβ+ and CU Aβ+ groups, in regions of the bilateral posterior insula, cuneus, superior temporal gyrus, and right fusiform gyrus (Figure 2).

In a subsequent analysis, we further stratified CU participants according to both amyloid and tau status.^3^ By using te‐ASL, we found that the levels of mean CBF presented a decreasing trend across more advanced stages with respect to the CU A−T− group (CU A+T− = −4.86%, *p* = 0.307, and CU A+T+ = −9.30%, *p* = 0.016) and to the CU A+T− group (CU A+T+ = −4.44%, *p *= 0.254, and CI Aβ+ = −17.00%, *p* = 0.026) (Figure S4).

### 3.5 Associations of CBF with biomarkers

**FIGURE 3:**
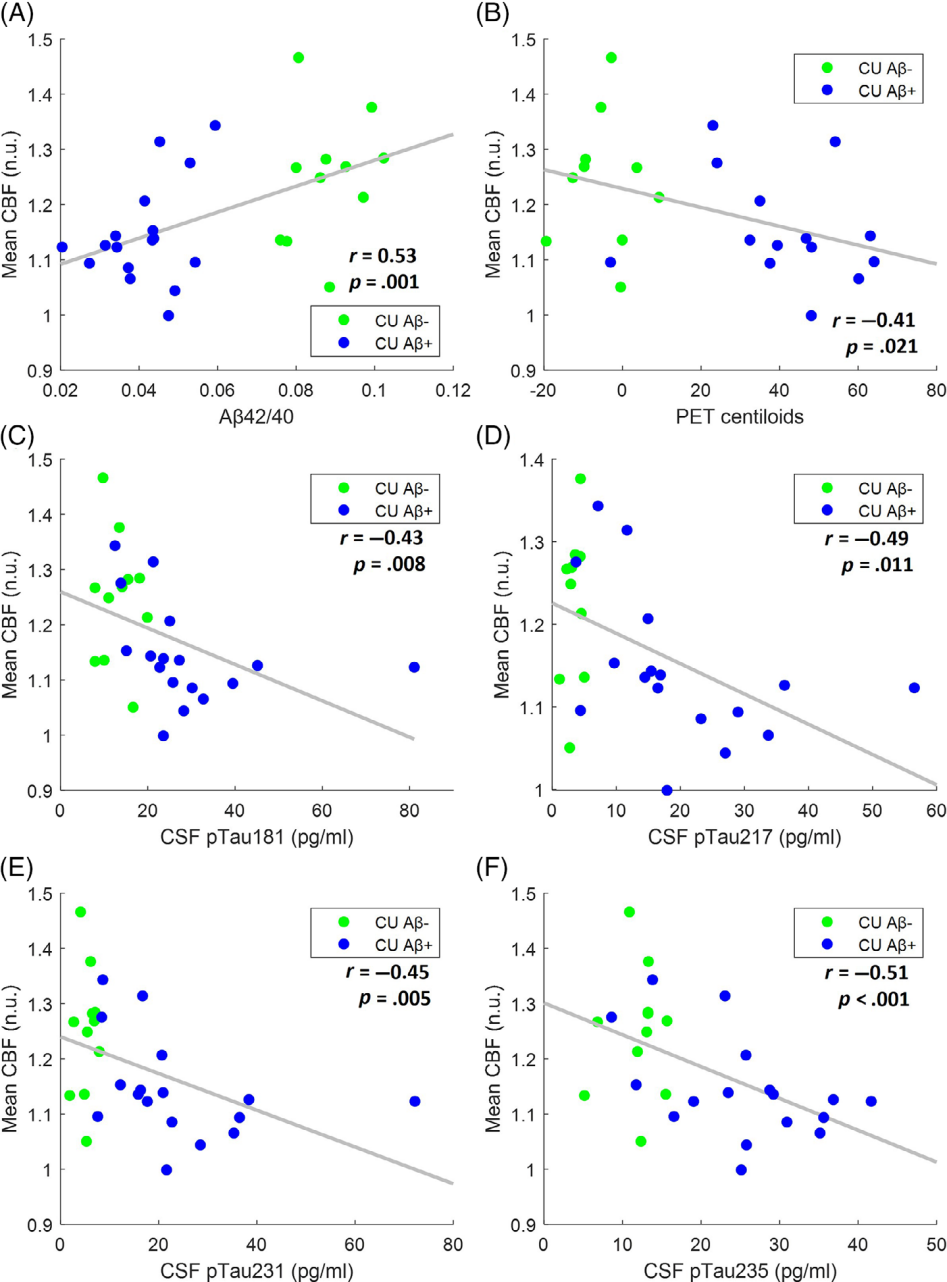
Association of CBF within a first data‐driven mask with levels of Aβ and tau proteins in CU individuals. The first data‐driven mask consisted of areas of the brain where te‐ASL revealed significantly reduced CBF in CI Aβ+ individuals, in comparison with CU Aβ− individuals. Scatterplots representing the association of mean CBF with (A) CSF Aβ42/40 (11 CU Aβ− and 17 CU Aβ+ subjects); (B) Aβ PET Centiloids (10 CU Aβ− and 14 CU Aβ+ subjects); (C) CSF pTau181 (11 CU Aβ− and 17 CU Aβ+ subjects); (D) CSF pTau217 (10 CU Aβ− and 17 CU Aβ+ subjects); (E) CSF pTau231 (11 CU Aβ− and 17 CU Aβ+ subjects); and (F) CSF pTau235 (11 CU Aβ− and 17 CU Aβ+ subjects). FDR‐corrected *p*‐values are reported in the “Results” section. Lower CBF was associated with lower levels of Aβ42/40, higher levels of Aβ PET Centiloids, and higher levels of all four tau proteins. Aβ, amyloid‐beta; Aβ−, normal levels of Aβ proteins; Aβ+, altered levels of Aβ proteins; ASL, arterial spin labeling; CBF, cerebral blood flow; CI, cognitively impaired; CSF, cerebrospinal fluid; CU, cognitively unimpaired; FDR, false discovery rate; n.u., normalized units; PET, positron emission tomography; te, time‐encoded.

**FIGURE 4:**
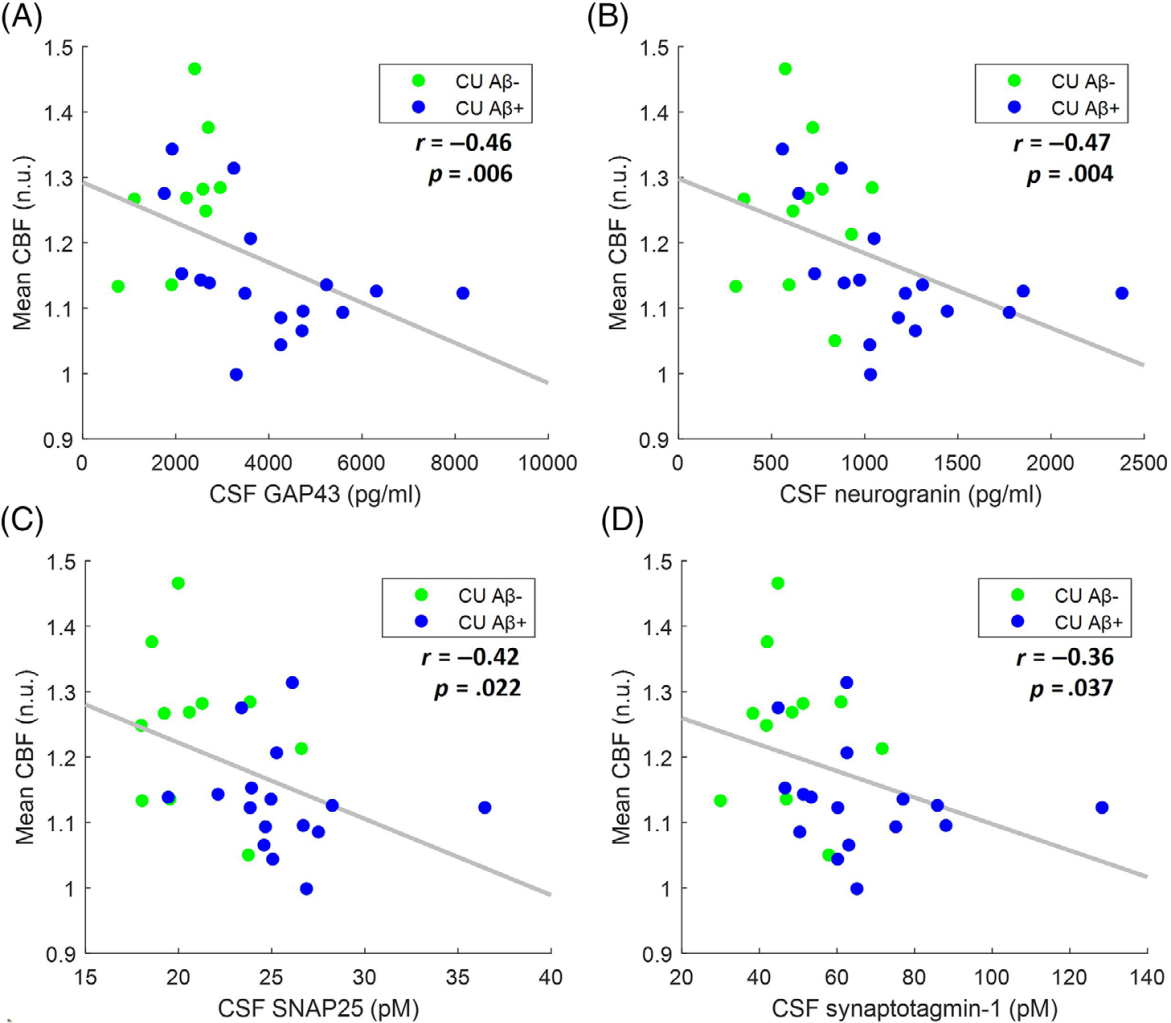
Association of CBF within a first data‐driven mask with biomarkers of synaptic dysfunction in CU individuals. The first data‐driven mask consisted of areas of the brain where te‐ASL revealed significantly reduced CBF in CI Aβ+ individuals, in comparison with CU Aβ− individuals. Scatterplots representing the association of mean CBF with (A) CSF GAP43 (9 CU Aβ− and 17 CU Aβ+ subjects); (B) CSF neurogranin (11 CU Aβ− and 17 CU Aβ+ subjects); (C) CSF SNAP25 (11 CU Aβ− and 16 CU Aβ+ subjects); and (D) CSF synaptotagmin‐1 (11 CU Aβ− and 16 CU Aβ+ subjects). FDR‐corrected *p*‐values are reported in the “Results” section. Lower CBF was associated with higher levels of CSF GAP43, neurogranin, and SNAP25. Aβ, amyloid beta; Aβ−, normal levels of Aβ proteins; Aβ+, altered levels of Aβ proteins; ASL, arterial spin labeling; CBF, cerebral blood flow; CI, cognitively impaired; CSF, cerebrospinal fluid; CU, cognitively unimpaired; FDR, false discovery rate; GAP43, growth‐associated protein 43; n.u., normalized units; SNAP25, synaptosomal‐associated protein 25; te, time‐encoded.

**FIGURE 5:**
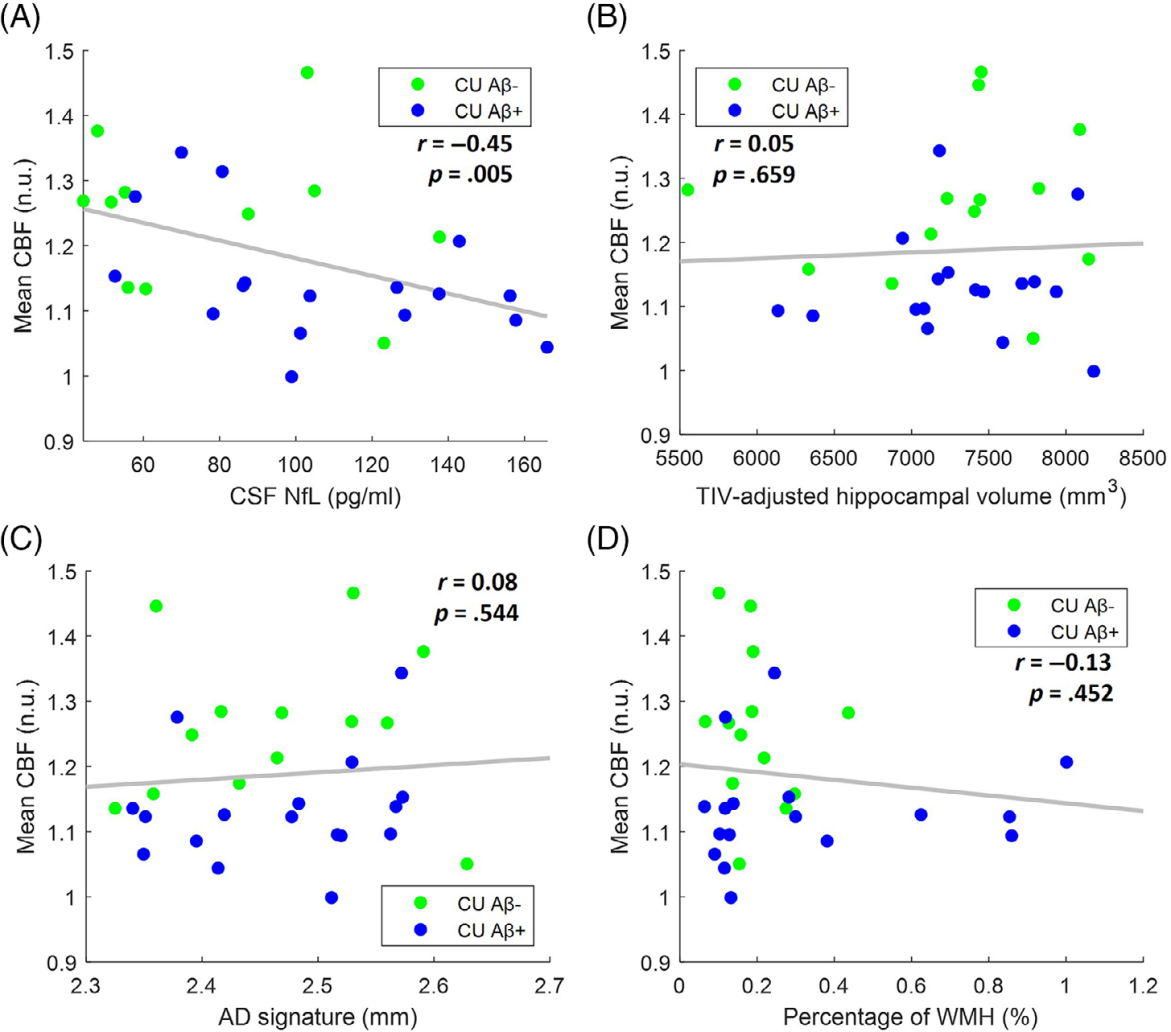
Association of CBF within a first data‐driven mask with biomarkers of neurodegeneration and cerebrovascular disease in CU individuals. The first data‐driven mask consisted of areas of the brain where te‐ASL revealed significantly reduced CBF in CI Aβ+ individuals, in comparison with CU Aβ− individuals. Scatterplots representing the association of mean CBF with (A) CSF NfL (11 CU Aβ− and 17 CU Aβ+ subjects); (B) TIV‐adjusted hippocampal volume (13 CU Aβ− and 17 CU Aβ+ subjects); (C) AD signature (13 CU Aβ− and 17 CU Aβ+ subjects); and (D) percentage of WMH (13 CU Aβ− and 17 CU Aβ+ subjects). FDR‐corrected *p*‐values are reported in the “Results” section. Lower CBF was associated with higher levels of CSF NfL. Aβ, amyloid beta; Aβ−, normal levels of Aβ proteins; Aβ+, altered levels of Aβ proteins; AD, Alzheimer's disease; ASL, arterial spin labeling; CBF, cerebral blood flow; CI, cognitively impaired; CSF, cerebrospinal fluid; CU, cognitively unimpaired; NfL, neurofilament light; n.u., normalized units; te, time‐encoded; TIV, total intracranial volume; WMH, white matter hyperintensities.

Finally, to study CBF association with markers of AD, synaptic dysfunction, neurodegeneration, and cognition in all CU individuals, we investigated it in both the CU Aβ+ and CU Aβ− groups in the first data‐driven mask (areas of the brain where te‐ASL revealed significantly reduced CBF in CI Aβ+ individuals, in comparison with CU Aβ− individuals). We found that lower CBF was associated with lower levels of CSF Aβ42/40 (*r *= 0.53; *p *= 0.001; *p*_FDR_ = 0.010) and with higher levels of Aβ PET Centiloids (*r *= −0.41; *p* = 0.021; *p*_FDR_ = 0.043) (Figure 3A,B). Moreover, lower CBF was associated with higher levels of all four tau proteins studied in the CSF: pTau181 (*r *= −0.43; *p *= 0.008; *p*_FDR_ = 0.023); pTau217 (*r *= −0.49; *p *= 0.011; *p*_FDR_ = 0.028); pTau231 (*r *= −0.45; *p *= 0.005; *p*_FDR_ = 0.019); and pTau235 (*r *= −0.51; *p *< 0.001; *p*_FDR_ = 0.008) (Figure 3C–F). Lower CBF was also associated with higher levels of three of the four biomarkers of synaptic dysfunction studied: GAP43 (*r *= −0.46; *p *= 0.006; *p*_FDR_ = 0.019), neurogranin (*r *= −0.47; *p *= 0.004; *p*_FDR_ = 0.019), and SNAP25 (*r *= −0.42; *p *= 0.022; *p*_FDR_ = 0.043), but not synaptotagmine‐1 (*r *= −0.36; *p = *.037; *p*_FDR_ = 0.061) (Figure 4). Finally, we observed that lower CBF was associated with higher levels of the neurodegeneration biomarker CSF NfL (*r *= −0.45; *p *= 0.005; *p*_FDR_ = 0.019) (Figure 4A), but not with imaging neurodegeneration markers TIV‐adjusted hippocampal volume and AD signature (Figure 5B,C). However, in a secondary analysis examining the association of CBF with hippocampal volume and AD signature that included both CU and CI individuals, we observed reduced levels of CBF in individuals with lower values of hippocampal volume (*r* = 0.44; *p* < 0.001) and AD signature (*r* = 0.41; *p* < 0.003) (Figure S5). We also ruled out neurovascular disease as a potential cause of CBF reduction as we found no association between lower CBF and the percentage of WMH (Figure 5D).

To confirm the results for the association of CBF in the first data‐driven mask with the aforementioned markers in CU individuals, we performed the same analyses in the a priori mask (areas of the brain associated with CBF reduction in AD, as previously reported^11^) and in the second data‐driven mask (areas of the brain where te‐ASL revealed significantly reduced CBF in CI Aβ+ individuals, in comparison with CU Aβ− individuals; Figures S6–S11). Generally, correlation values were lower in the a priori mask analysis than in the first data‐driven mask, and *p*values were < 0.05 only for Aβ42/40 and pTau235. With respect to the second data‐driven mask analysis, higher correlation values were observed when compared to the first data‐driven mask. In addition, a strong association was found between mean CBF and synaptotagmine‐1 (*r* = −0.49; *p* = 0.022; *p*_FDR_ = 0.040), a relationship that was not evident in the first data‐driven mask analysis.

Finally, we analyzed for the CU Aβ+ and CU Aβ− groups the association of CBF in the three masks with cognition markers and only found a significant association between lower CBF and lower language composite score (*r *= 0.36; *p *= 0.026; *p*_FDR_ = 0.047) in the first data‐driven mask (Figure S12–S14). However, in a secondary analysis examining the association of CBF with MMSE score that included both CU and CI individuals, we observed reduced levels of CBF in individuals with lower MMSE scores (Figure S15; *r* = 0.58; *p* < 0.001).

## 4 DISCUSSION

We have provided evidence that CBF reduction is present in CU Aβ+ individuals; therefore, it is an earlier event in the AD pathological cascade than previously thought, spanning preclinical stages. We detected such early CBF reductions using te‐ASL, which we demonstrated to be more sensitive than single‐PLD ASL (PLD = 2000 ms) at detecting CBF changes across the AD continuum. Moreover, we showed that CBF reduction was associated with biomarkers of AD, synaptic dysfunction, and neurodegeneration in CU individuals.

Using te‐ASL, we observed that low CBF was associated with reduced levels of CSF Aβ42/40 and high levels of Aβ PET Centiloids in CU individuals, in line with studies in CI Aβ+ individuals.^4^, ^33^, ^53^ A previous report showed that CBF was associated with Aβ biomarkers in CU individuals^6^; however, most other studies failed to detect this association,^11^, ^12^, ^13^, ^14^ possibly because they used other brain imaging techniques. Notably, using single‐PLD ASL, like a recent study by Ahmadi and colleagues (2023),^11^ we also failed to detect a link between CBF and Aβ biomarkers in CU individuals.

A voxel‐wise analysis in CU Aβ+ individuals revealed reduced CBF in several brain regions, including the cuneus, superior temporal gyrus, right fusiform gyrus, and lateral frontal regions. These areas were previously reported to exhibit reduced CBF in AD individuals^11^ and were also evident in our CI Aβ+ sample, with the exception of the lateral frontal regions. While a voxel‐wise analysis did not detect pronounced CBF reductions in lateral frontal regions for CI Aβ+ individuals, a subsequent post hoc analysis focused on the middle frontal gyrus presented significant reductions in this group when considering te‐ASL‐derived CBF maps (Figure S15). The absence of marked CBF reductions in these areas at the voxel level may be attributed to the limited statistical power in our study but also to influences from brain activity and mood state, which are known to influence CBF in frontal areas.^54^

Our findings of lower CBF in CU Aβ+ individuals can be reconciled with previous findings of a positive association of CBF with early amyloid pathology^15^ since biphasic behaviors are commonly observed in preclinical AD stages for other imaging biomarkers like cortical thickness or glucose consumption.^55^ Note that the average amyloid CL value in Padrela et al.^15^ was 12, whereas in our CU Aβ+ group it was over 40. Therefore, it is plausible that CBF increases early in the preclinical AD continuum in response to amyloid pathology and decreases later on with the presence of tau abnormalities. Further studies across the preclinical AD continuum are needed to confirm this hypothesis.

Our results suggest that the increased sensitivity of te‐ASL at detecting early and subtle CBF changes may be primarily attributed to accounting for between‐subject factors (eg, sex, age) and intracerebral differences in ATT. Notably, no significant ATT differences were observed between groups. Additional sources of discrepancy may have arisen from the characteristics of the studied cohort, such as the load of AD pathology, percentage of *APOE‐*ε4 carriers, and presence of cerebrovascular disease, which were not examined in any of the aforementioned studies. To exclude potential underlying causes other than AD pathophysiology for the differences in CBF between CU Aβ− and CU Aβ+ individuals, we analyzed the *APOE* genotypes. It was shown that CU carriers of the *APOE‐*ε*4* allele had increased CBF.^56^ In our cohort, a higher percentage of *APOE*‐*ε4* carriers were found in the CU Aβ+ group, as expected, since *APOE*‐*ε4* is a strong risk factor for Aβ accumulation. Therefore, we ruled out that *APOE‐ε4* could drive the higher levels of CBF in the CU Aβ− group. Moreover, to exclude cerebrovascular disease as a factor underlying CBF reductions observed in the CU Aβ+ group, in CU individuals, we looked at the percentage of WMH, a widely recognized hallmark of small vessel disease.^57^ We observed no significant differences in the volume of WMH between CU Aβ− and Aβ+ individuals and found no association between CBF and WMH. In summary, the observed reduction in CBF in the CU Aβ+ group was likely driven by AD pathophysiology.

For the first time, we showed associations of lower CBF with higher levels of biomarkers of tau pathophysiology in CU individuals, in line with what has been reported in CI Aβ+ individuals.^11^ However, the CBF association with tau proteins in CSF and blood does not seem completely independent from its association with Aβ biomarkers.^11^, ^58^ Unfortunately, the lack of available tau PET imaging in this study prevented us from definitively establishing an association of lower CBF with neurofibrillary tau pathology.

We also found for the first time in CU individuals an association of lower CBF with higher levels of CSF NfL, but not with hippocampal volume and AD signature, all biomarkers of neurodegeneration.^30^, ^55^ Hippocampal volume and AD signature were found to be positively associated with CBF only when pooling CU and CI individuals, which is in line with previous findings (Figure S5).^11^, ^59^, ^60^ It is important to note that CSF NfL measures the present rate of axonal injury. In contrast, the imaging markers reflect the accumulated cerebral volumetric changes over time, which, along the preclinical AD continuum, show a non‐linear trajectory.^31^ Furthermore, the levels of CSF NfL in CU Aβ+ individuals substantially overlapped with those of the CU Aβ− group. This suggests that our CU sample may be at a very early stage in the AD continuum, thereby demonstrating an association with the current rate of neurodegeneration but not yet with accumulated neurodegeneration.^61^, ^62^

Lastly, when pooling CU and CI individuals together, we found reduced CBF in individuals exhibiting lower cognitive performance, as indicated by their MMSE scores, aligning with prior research.^17^, ^63^ However, we could not detect any significant associations between CBF and cognition when examining CU individuals only, apart from language composites in the second data‐driven mask. This lack of association can be attributed to the limited range of cognitive decline in our CU sample and the fact that the CU Aβ+ participants were at an early stage in the AD continuum.

This study had several limitations. First, it was a cross‐sectional study with a relatively small and homogeneous sample; therefore, we could not untangle whether decreased CBF triggered the chain of pathological events in AD or whether it was a consequence of Aβ and tau pathologies. Further, we cannot draw conclusions regarding the potential influence of additional factors contributing to diversity. Larger longitudinal studies are under way to specifically address these aspects and expand our findings. Second, age and sex were not balanced among the study groups, which could have resulted in biased comparisons. However, age and sex differences are well known and were accounted for in the statistical analyses. Third, 13 out of the 24 CU Aβ− participants did not have CSF biomarkers available, so we could not exclude the possibility that some were CU Aβ+. Indeed, an estimated 20% of CU people in the age range of the participants in our study were positive for AD biomarkers^26^; therefore, we may have misclassified up to two to three CU Aβ− participants. However, it is unlikely that such an eventual small variation would significantly affect our results. Another limitation is that we measured CBF, CSF biomarkers, and cognitive scores at different time points. However, since the progression of AD is protracted, the difference in time between measurements is not expected to have a major impact on the interpretation of the results. Furthermore, the lack of tau PET does not allow us to firmly state the association of lower CBF with neurofibrillary tau pathology in CU individuals.^64^, ^65^ However, this association seems plausible since it has been firmly established in CI Aβ+ individuals. Another limitation of the study was that single‐PLD ASL was derived from the te‐ASL data to be compared to the latter. Although it was previously shown that this results in a similar temporal signal‐to‐noise ratio as single‐PLD ASL,^66^ additional studies with a direct comparison to single‐PLD ASL would be informative. Finally, in our analysis, we did not apply correction for partial volume effect, which can confound the estimation of perfusion, especially in individuals with atrophy.^67^ We omitted partial volume effect correction as correction methods typically amplify noise and this might have differentially affected the two ASL techniques and bias their comparison. However, we do not believe partial volume effects interfere with the comparison of ASL methods, as single‐PLD ASL and te‐ASL are affected in an identical manner. Moreover, given that we did not observe an association of CBF with measures of brain atrophy in CU individuals, it is unlikely that the CBF hypoperfusion observed in CU Aβ+ individuals was driven by partial volume effects.

In conclusion, CBF reduction occurs earlier in the AD continuum than previously thought, spanning preclinical stages. Also, we proved that te‐ASL was more sensitive than single‐PLD ASL in measuring CBF alterations in AD. Finally, we showed that lower CBF was associated with altered markers of AD, synaptic dysfunction, and neurodegeneration in CU individuals.

## CONFLICT OF INTEREST STATEMENT

Javier Sánchez‐Gonzalez and Paula Montesinos are Philips Healthcare Iberia employees. Gwendlyn Kollmorgen is a full‑time employee of Roche Diagnostics GmbH, Penzberg, Germany. Clara Quijano‐Rubio is a full‐time employee of Roche Diagnostics International Ltd, Rotkreuz, Switzerland. José Luis Molinuevo is now an employee of H. Lundbeck A/S. Albert Puig‐Pijoan has served on advisory boards for Schwabe Farma Iberica. Marc Suárez‐Calvet has given lectures in symposia sponsored by Roche Diagnostics, S.L.U, Roche Farma, S.A, Eli Lilly, and Amirall; he has served as a consultant and on advisory boards for Roche Diagnostics International Ltd and Grifols S.L.; he received a grant under a project funded by Roche Diagnostics International Ltd; and he received in‐kind support for research from Roche Diagnostics International Ltd, Avid Radiopharmaceuticals, Inc., Eli Lilly, and Janssen Research & Development. All payments were made to the institution (BBRC). Henrik Zetterberg has served on scientific advisory boards and/or as a consultant for Abbvie, Acumen, Alector, Alzinova, ALZPath, Annexon, Apellis, Artery Therapeutics, AZTherapies, Cognito Therapeutics, CogRx, Denali, Eisai, Merry Life, Nervgen, Novo Nordisk, Optoceutics, Passage Bio, Pinteon Therapeutics, Prothena, Red Abbey Labs, reMYND, Roche, Samumed, Siemens Healthineers, Triplet Therapeutics, and Wave, has given lectures at symposia sponsored by Alzecure, Biogen, Cellectricon, Fujirebio, Lilly, and Roche, and is a co‐founder of Brain Biomarker Solutions in Gothenburg AB (BBS), which is a part of the GU Ventures Incubator Program (outside submitted work). Juan Domingo Gispert has served as a consultant for Roche Diagnostics and Prothena Biosciences; he has given lectures at symposia sponsored by General Electric, Philips, Esteve, Life‐MI, and Biogen; and he received research support from GE Healthcare, Roche Diagnostics, and Hoffmann‐La Roche. The Roche NeuroToolKit is a panel of exploratory prototype assays designed to robustly evaluate biomarkers associated with key pathologic events characteristic of AD and other neurological disorders; it is used for research purposes only and not approved for clinical use. Elecsys pTau181 CSF assay is approved for clinical use. COBAS and ELECSYS are trademarks of Roche. All other product names and trademarks are the property of their respective owners. All other authors report that they have no relevant relationships or conflicts of interest regarding the contents of this paper. Author disclosures are available in the Supporting information.

## CONSENT STATEMENT

All participants provided written informed consent.

## Supporting information

Supporting Information

Supporting Information
