# Supplementary material for: Time‐encoded ASL reveals lower cerebral blood flow in the early AD continuum
Source: Alzheimers Dement. 2024 Jul 3;20(8):5183–97. doi: 10.1002/alz.14059 (PMC11350027; doi:10.1002/alz.14059)
Supplement: Supplementary file 1 — Supporting Information [file ALZ-20-5183-s002.pdf]

SUPPLEMENTARY DATA

TABLE S1. CSF biomarkers for CU Aβ- and CU Aβ+ participants.

	CU Aβ- (n = 11)	CU Aβ+ (n = 17)	CU Aβ- vs CU Aβ+ (*p* value)
**CSF Aβ42/40**	0.088 (0.009)	0.041 (0.010)	<0.001
**CSF pTau181, pg/ml**	218.9 (81.8)	918.3 (604.0)	<0.001
**CSF pTau217, pg/ml**	3.4 (1.2)	19.9 (13.4)	<0.001
**CSF pTau231, pg/ml**	5.3 (1.9)	23.5 (15.7)	<0.001
**CSF pTau235, pg/ml**	11.9 (3.3)	25.4 (9.3)	<0.001
**CSF NfL, pg/ml**	79.3 (33.3)	107.7 (36.0)	0.043
**CSF GAP43, pg/ml**	2144.5 (751.5)	3997.6 (1696.2)	<0.001
**CSF neurogranin, pg/ml**	676.0 (222.9)	1188.8 (467.7)	<0.001
**CSF SNAP25, pM**	20.9 (2.8)	25.6 (3.6)	<0.001
**CSF synaptotagmin-1, pM**	48.5 (11.6)	67.2 (20.8)	0.007

Data are expressed as mean (SD) for all measurements. The *p* values were computed with *t*-test. CSF biomarkers were available for 11 out of 24 CU Aβ- and 17 out of 18 CU Aβ+ participants. Aβ: amyloid-beta; CSF: cerebrospinal fluid; CU: cognitively unimpaired; NfL: neurofilament light; GAP43: growth-associated protein-43; SNAP25: synaptosomal-associated protein-25.

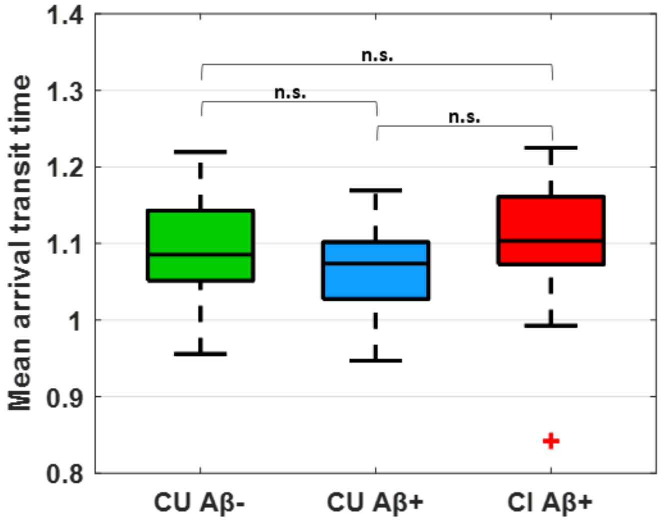

FIGURE S1. Mean ATT measured using te-ASL within the *a priori* mask. The three groups, CU Aβ-, CU Aβ+ and CI Aβ+, presented similar values of mean ATT.

Aβ: amyloid-beta; A-: normal levels of Aβ proteins; A+: altered levels of Aβ proteins; ATT: arterial transit time; CI: cognitively impaired; CU: cognitively unimpaired.

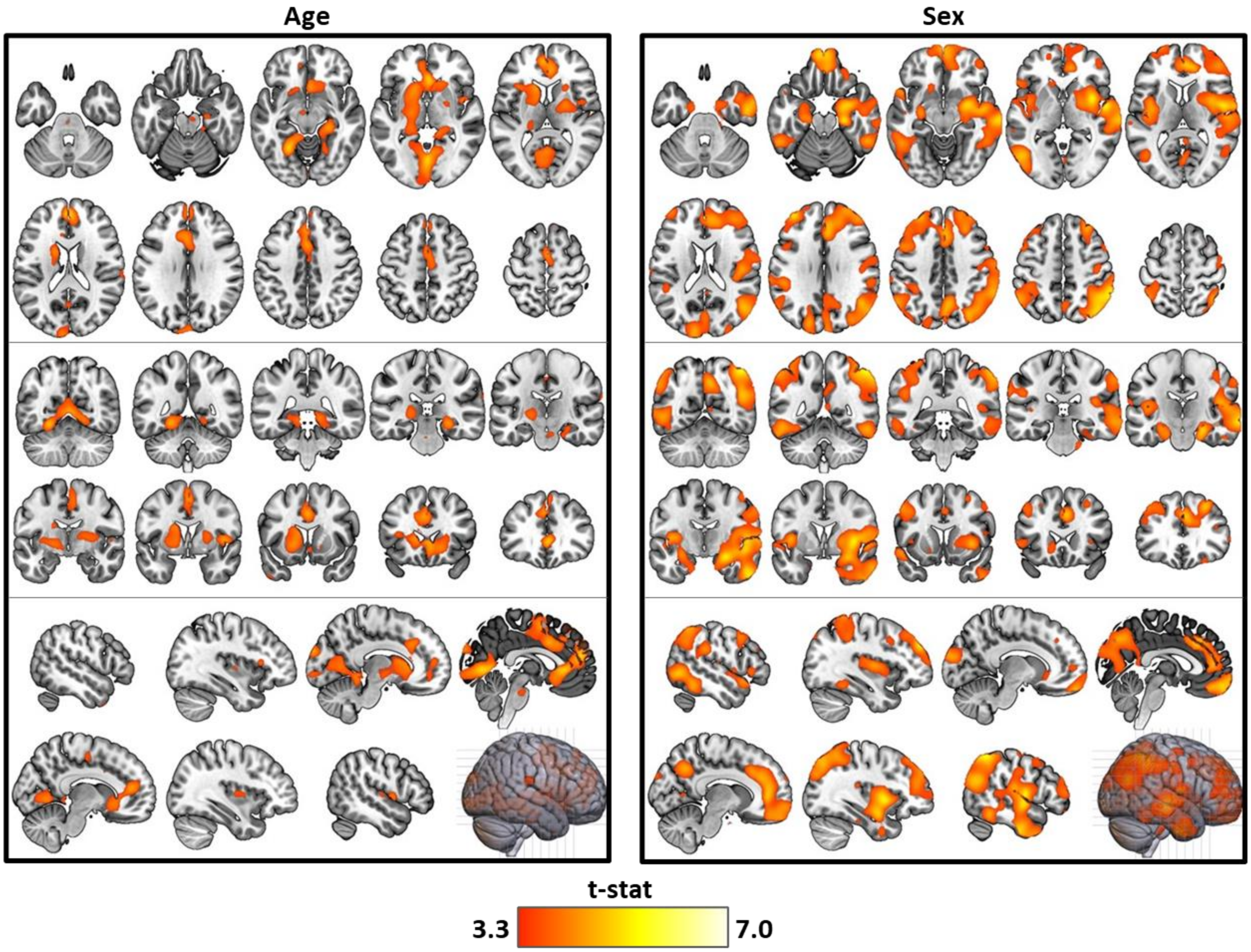

FIGURE S2. Effect of age and sex on arterial transit time (ATT) as measured with te-ASL. Age was positively associated with ATT in several regions, including the anterior cingulate, medial frontal gyrus, precuneus, lingual gyrus, and bilateral caudate, putamen and thalamus. In addition, compared to females, males presented higher ATT in widespread regions of gray matter such as in middle frontal gyrus, anterior cingulate, posterior insula, precuneus, inferior parietal lobe, angular gyrus, and middle temporal gyrus. No significant differences were observed across diagnostic groups.

ATT: arterial transit time.

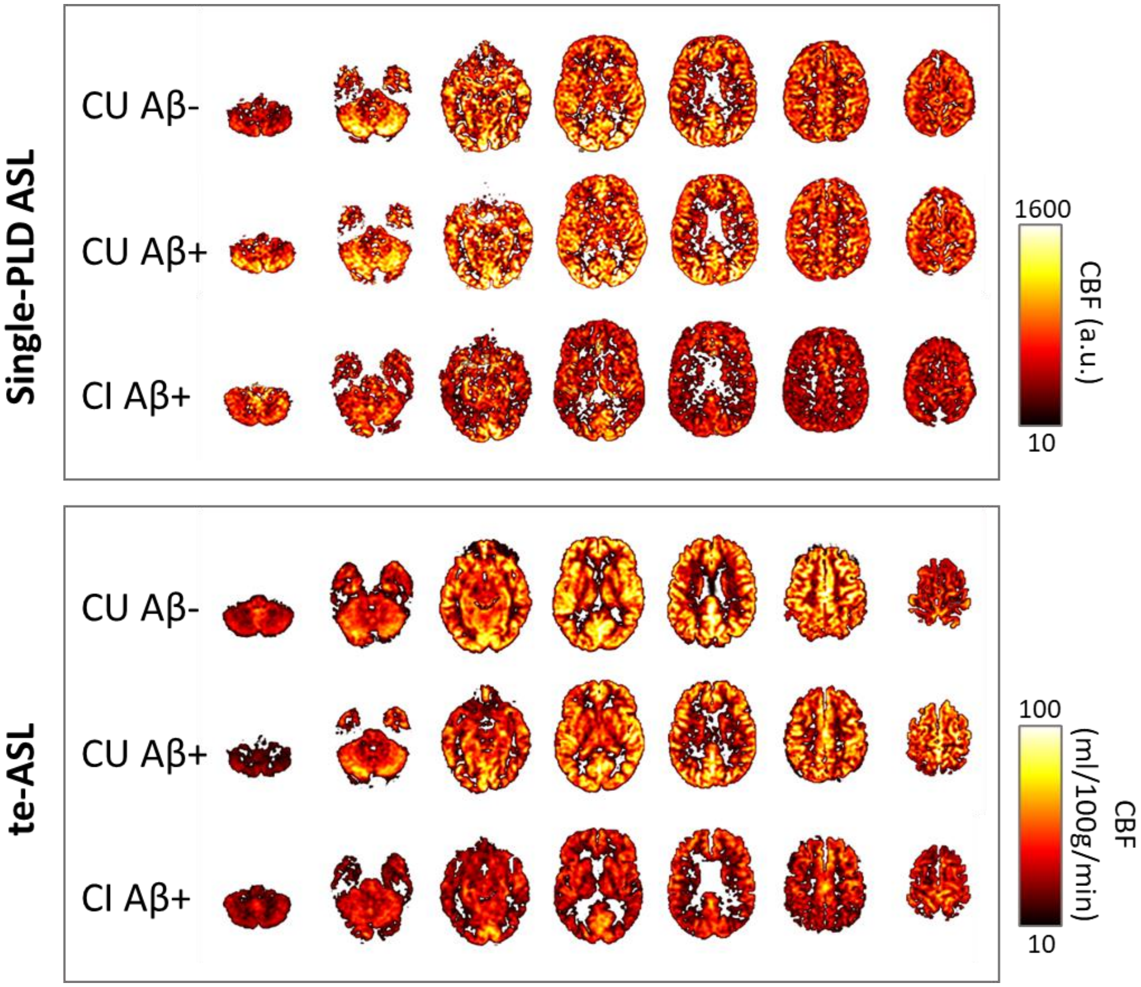

FIGURE S3. Representative subject-level CBF maps for each diagnosis group obtained with single-PLD ASL (top) and te-ASL (bottom). The CBF maps presented here correspond to the CBF maps derived in native space by decoding the ASL-signal images (see Section 2.2.). Note that, prior to any analysis, the maps underwent registration to MNI space, spatial smoothing (12 mm FWHM), and normalization to the mean value in the cerebellar gray matter. Consistent with the group-level findings, we observe lower CBF in the CI Aβ+ subject compared to the two CU subjects.

Aβ: amyloid-beta; A-: normal levels of Aβ proteins; A+: altered levels of Aβ proteins; CBF: cerebral blood flow; CI: cognitively impaired; CU: cognitively unimpaired.

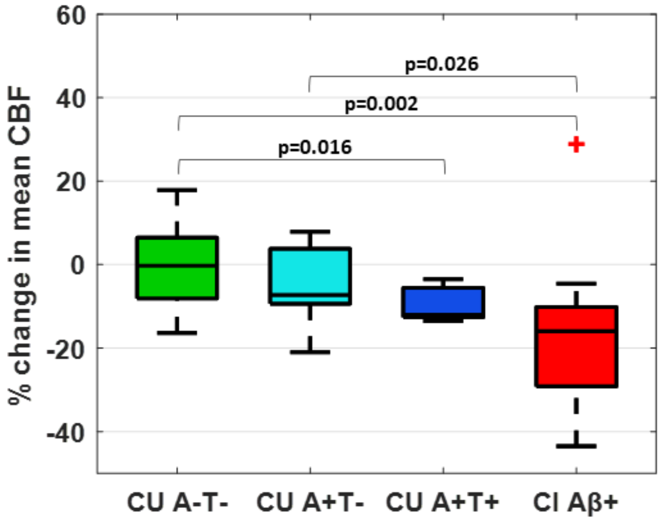

FIGURE S4. Percentage change in mean CBF measured using te-ASL within the *a priori* mask, in relation to Aβ and tau statuses.

The *a priori* mask consisted of areas of the brain associated with CBF reduction in AD, as previously reported [11]. The percentage change in mean CBF is represented with respect to the CU A-T- group. Individuals were stratified into four groups on the basis of the Aβ and tau statuses (A+ if CSF Aβ42/40 was < 0.071; and T+ if CSF pTau181 was > 24 pg/ml [2]): CU A-T- ($n = 11$), CU A+T- ($n = 8$), CU A+T+ ($n = 9$), and CI Aβ ($n = 17$). Mean CBF presented a decreasing trend across more advanced stages of AD. The red cross above the last boxplot indicates an outlier.

Aβ: amyloid-beta; A-: normal levels of Aβ proteins; A+: altered levels of Aβ proteins; CBF: cerebral blood flow; CI: cognitively impaired; CU: cognitively unimpaired; T-: normal levels of tau proteins; T+: accumulation of tau proteins.

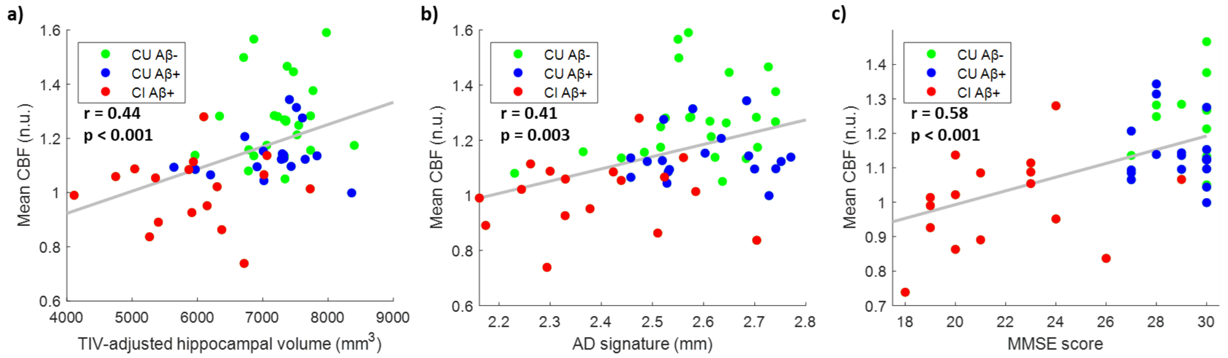

FIGURE S5: Association of CBF within a first data-driven mask with TIV-adjusted hippocampal volume, AD signature, and MMSE scores in all individuals.

The first data-driven mask consisted of areas of the brain where te-ASL revealed significantly reduced CBF in CI Aβ+ individuals, in comparison with CU Aβ- individuals. Scatterplot representing the association of mean CBF with (a) TIV-adjusted hippocampal volume (24 CU Aβ-, 18 CU Aβ+ subjects and 17 CI Aβ+ subjects), (b) AD signature (24 CU Aβ-, 18 CU Aβ+ subjects and 17 CI Aβ+ subjects) and (c) MMSE score (11 CU Aβ-, 18 CU Aβ+ subjects and 17 CI Aβ+ subjects) in both CU and CI subjects. Note that the hippocampal volume and AD signature values presented in this figure were derived from T1w images acquired from all 59 participants during the ASL MRI session. In contrast, the equivalent values presented in Figure 5 were obtained from T1w images collected for a subset of CU individuals during the structural MRI session. The T1w images acquired during the structural MRI session are theoretically better suited for computing the hippocampal volume and AD signature due to their isotropic voxel size and higher resolution; however, in practice, the differences in estimated values between the two series of T1w images were negligible. Lower CBF was associated with a lower hippocampal volume, AD signature and MMSE score.

Aβ: amyloid-beta; Aβ-: normal levels of Aβ proteins; Aβ+: altered levels of Aβ proteins; ASL: arterial spin labeling; CBF: cerebral blood flow; CU: cognitively unimpaired; n.u.: normalized units; MMSE: Mini-Mental State Examination; te: time-encoded; TIV: total intracranial volume.

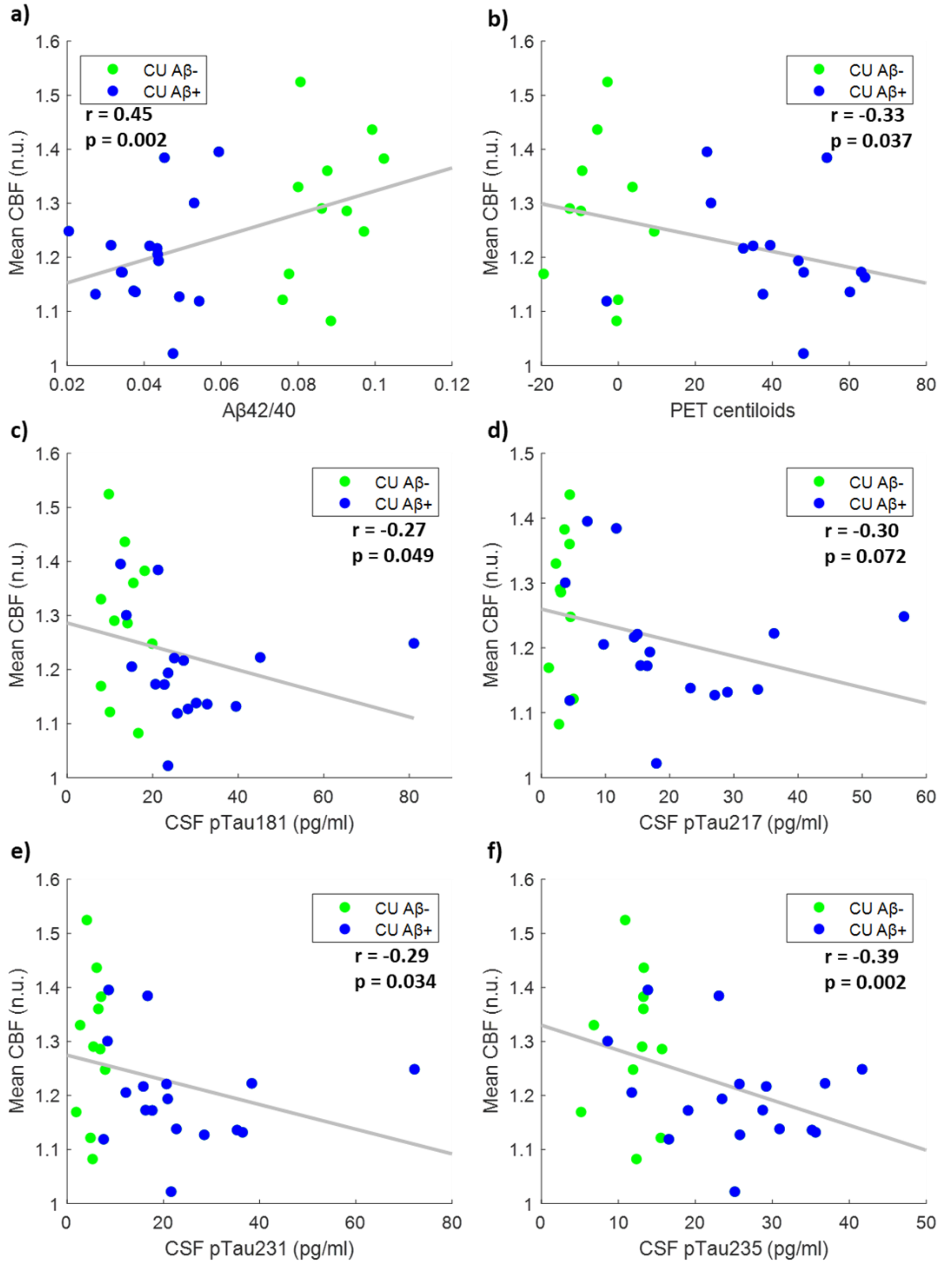

FIGURE S6: Association of CBF within an *a priori* mask with levels of Aβ and tau proteins in CU individuals.

The *a priori* mask consisted of areas of the brain associated with CBF reduction in AD, as previously reported [11]. Scatterplots representing the association of mean CBF with: **a)** CSF Aβ42/40 (11 CU Aβ- and 17 CU Aβ+ subjects); **b)** Aβ PET Centiloids (10 CU Aβ- and 14 CU Aβ+ subjects); **c)** CSF pTau181 (11 CU Aβ- and 17 CU Aβ+ subjects); **d)** CSF pTau217 (10 CU Aβ- and 17 CU Aβ+ subjects); **e)** CSF pTau231 (11 CU Aβ- and 17 CU Aβ+ subjects); and **f)** CSF pTau235 (11 CU Aβ- and 17 CU Aβ+ subjects). FDR-corrected *p*-values are reported in the "Results" section. Lower CBF was associated with lower levels of Aβ42/40 and with higher levels of pTau235.

Aβ: amyloid-beta; Aβ-: normal levels of Aβ proteins; Aβ+: altered levels of Aβ proteins; AD: Alzheimer's disease; CBF: cerebral blood flow; CSF: cerebrospinal fluid; CU: cognitively unimpaired; n.u.: normalized units; PET: Positron Emission Tomography.

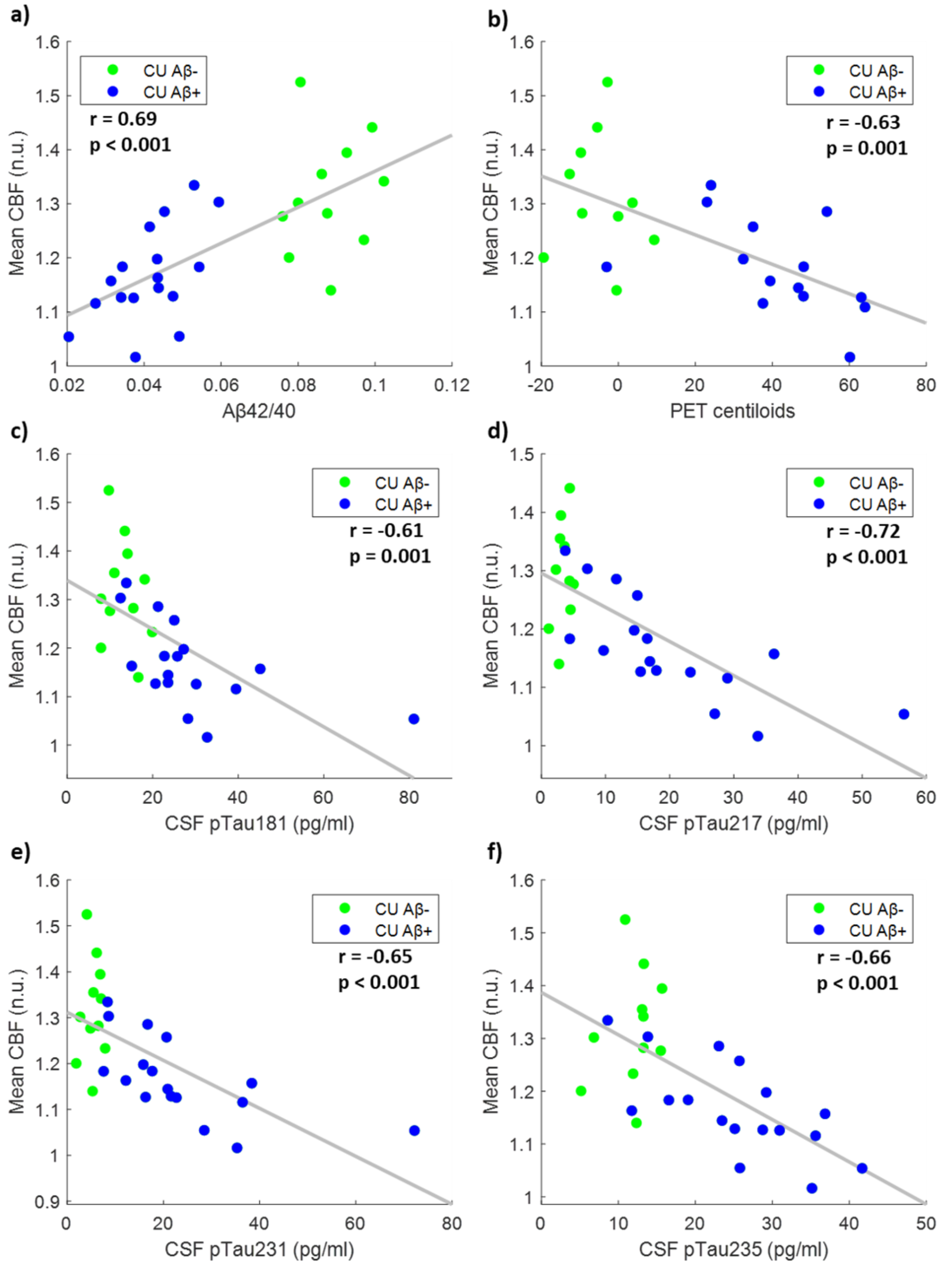

FIGURE S7: Association of CBF within a second data-driven mask with levels of Aβ and tau proteins in CU individuals.

The second data-driven mask consisted of areas of the brain where te-ASL revealed significantly reduced CBF in CU Aβ+ individuals, in comparison with CU Aβ- individuals. Scatterplots representing the association of mean CBF with **a)** CSF Aβ42/40 (11 CU Aβ- and 17 CU Aβ+ subjects), **b)** Aβ PET Centiloids (10 CU Aβ- and 14 CU Aβ+ subjects); **c)** CSF pTau181 (11 CU Aβ- and 17 CU Aβ+ subjects); **d)** CSF pTau217 (10 CU Aβ- and 17 CU Aβ+ subjects); **e)** CSF pTau231 (11 CU Aβ- and 17 CU Aβ+ subjects); and **f)** CSF pTau235 (11 CU Aβ- and 17 CU Aβ+ subjects). FDR-corrected *p*-values are reported in the "Results" section. Lower CBF was associated with lower levels of Aβ42/40 and with higher levels of Aβ PET Centiloids. Lower CBF was associated with higher levels of all four tau proteins.

Aβ: amyloid-beta; Aβ-: normal levels of Aβ proteins; Aβ+: altered levels of Aβ proteins; ASL: arterial spin labeling; CBF: cerebral blood flow; CSF: cerebrospinal fluid; n.u.: normalized units; PET: Positron Emission Tomography; te: time-encoded.

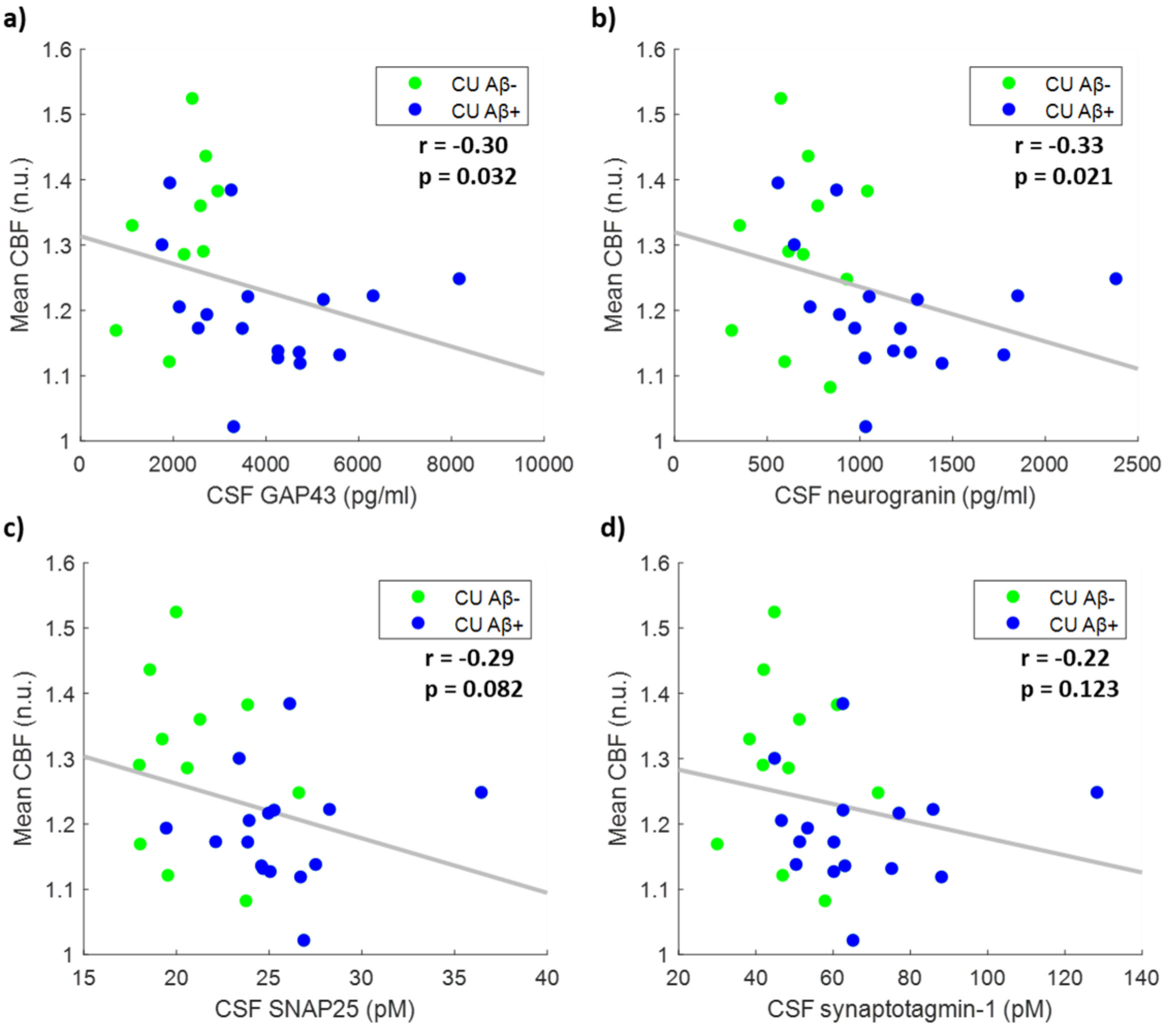

FIGURE S8: Association of CBF within an *a priori* mask with biomarkers of synaptic dysfunction in CU individuals.

The *a priori* mask consisted of areas of the brain associated with CBF reduction in AD, as previously reported [11]. Scatterplots representing the association of mean CBF with: **a)** CSF GAP43 (9 CU Aβ- and 17 CU Aβ+ subjects); **b)** CSF neurogranin (11 CU Aβ- and 17 CU Aβ+ subjects); **c)** CSF SNAP25 (11 CU Aβ- and 16 CU Aβ+ subjects); and **d)** CSF synaptotagmin-1 (11 CU Aβ- and 16 CU Aβ+ subjects). FDR-corrected *p*-values are reported in the "Results" section. Mean CBF was not associated with the levels of any biomarkers of synaptic dysfunction.

Aβ: amyloid-beta; Aβ-: normal levels of Aβ proteins; Aβ+: altered levels of Aβ proteins; ASL: arterial spin labeling; CBF: cerebral blood flow; CSF: cerebrospinal fluid; CU: cognitively unimpaired; GAP43: growth-associated protein 43; n.u.: normalized units; SNAP25: synaptosomal-associated protein 25; te: time-encoded.

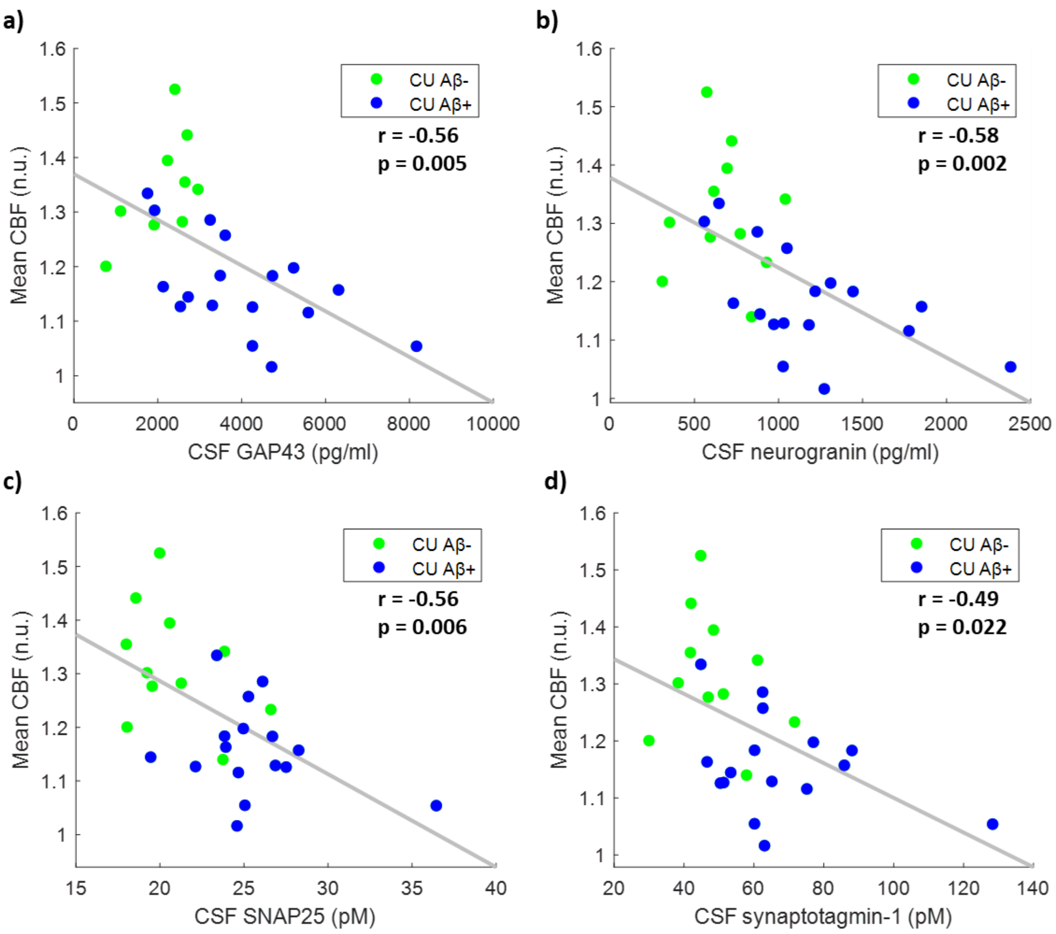

FIGURE S9: Association of CBF within a second data-driven mask with biomarkers of synaptic dysfunction in CU individuals.

The second data-driven mask consisted of areas of the brain where te-ASL revealed significantly reduced CBF in CU Aβ+ individuals, in comparison with CU Aβ- individuals. Scatterplots representing the association of mean CBF with: **a)** CSF GAP43 (9 CU Aβ- and 17 CU Aβ+ subjects); **b)** neurogranin (11 CU Aβ- and 17 CU Aβ+ subjects); **c)** SNAP25 (11 CU Aβ- and 16 CU Aβ+ subjects); and **d)** synaptotagmin-1; 11 CU Aβ- and 16 CU Aβ+ subjects). FDR-corrected *p*-values are reported in the "Results" section. Lower CBF was associated with higher levels of all four biomarkers of synaptic dysfunction.

Aβ: amyloid-beta; Aβ-: normal levels of Aβ proteins; Aβ+: altered levels of Aβ proteins; ASL: arterial spin labeling; CBF: cerebral blood flow; CSF: cerebrospinal fluid; CU: cognitively unimpaired; GAP43: growth-associated protein 43; n.u.: normalized units; SNAP25: synaptosomal-associated protein 25; te: time-encoded.

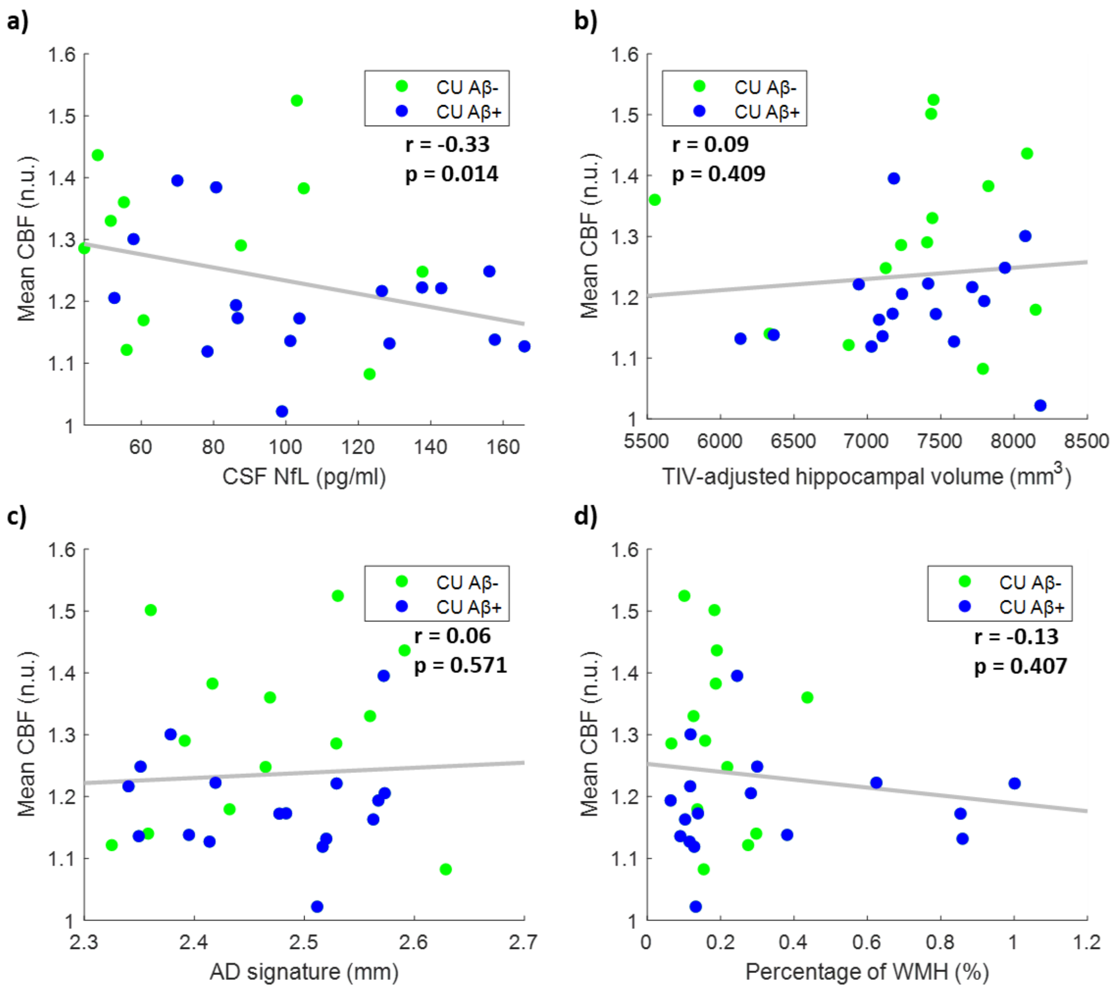

FIGURE S10: Association of CBF within an *a priori* mask with biomarkers of neurodegeneration and cerebrovascular disease in CU individuals.

The *a priori* mask consisted of areas of the brain associated with CBF reduction in AD, as previously reported [11]. Scatterplots representing the association of mean CBF with **a)** CSF NfL (11 CU Aβ- and 17 CU Aβ+ subjects); **b)** TIV-adjusted hippocampal volume (13 CU Aβ- and 17 CU Aβ+ subjects); **c)** AD signature (13 CU Aβ- and 17 CU Aβ+ subjects); and **d)** percentage of WMH (13 CU Aβ- and 17 CU Aβ+ subjects). FDR-corrected *p*-values are reported in the "Results" section. Mean CBF was not associated with the levels of any biomarker of neurodegeneration.

Aβ: amyloid-beta; Aβ-: normal levels of Aβ proteins; Aβ+: altered levels of Aβ proteins; AD: Alzheimer's disease; CBF: cerebral blood flow; CSF: cerebrospinal fluid; CU: cognitively unimpaired; NfL: neurofilament light; n.u.: normalized units; TIV: total intracranial volume; WMH: white matter hyperintensities.

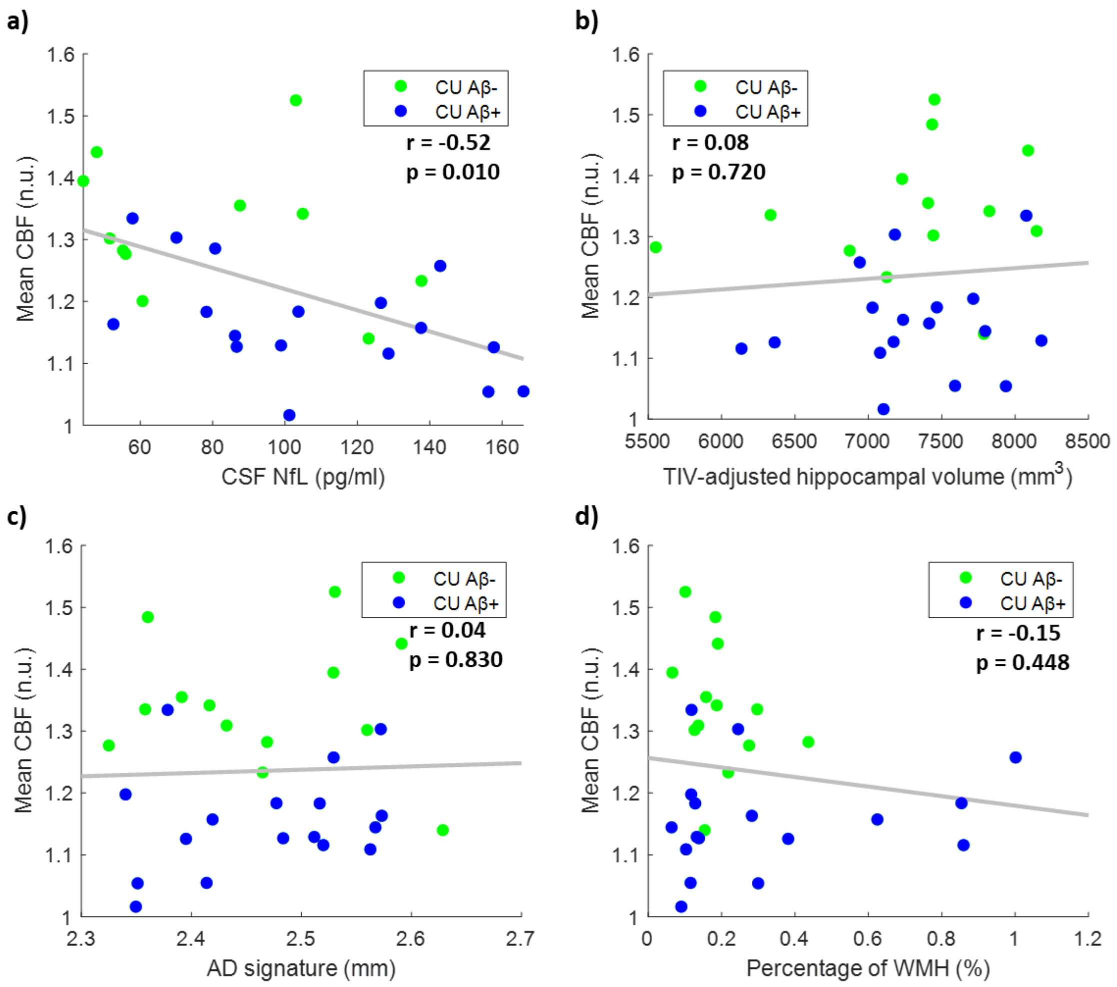

FIGURE S11: Association of CBF within a second data-driven mask with biomarkers of neurodegeneration and cerebrovascular disease in CU individuals.

The second data-driven mask consisted of areas of the brain where te-ASL revealed significantly reduced CBF in CU Aβ+ individuals, in comparison with CU Aβ- individuals. Scatterplots representing the association mean CBF with **a)** CSF NfL (11 CU Aβ- and 17 CU Aβ+ subjects); **b)** TIV-adjusted hippocampal volume (13 CU Aβ- and 17 CU Aβ+ subjects); **c)** AD signature (13 CU Aβ- and 17 CU Aβ+ subjects); and **d)** percentage of WMH (13 CU Aβ- and 17 CU Aβ+ subjects). FDR-corrected *p*-values are reported in the "Results" section. Lower CBF was associated with higher levels of CSF NfL (r = -0.52; p = 0.004).

Aβ: amyloid-beta; Aβ-: normal levels of Aβ proteins; Aβ+: altered levels of Aβ proteins; ASL: arterial spin labeling; CBF: cerebral blood flow; CSF: cerebrospinal fluid; CU: cognitively unimpaired; n.u.: normalized units; te: time-encoded; TIV: total intracranial volume; WMH: white matter hyperintensities.

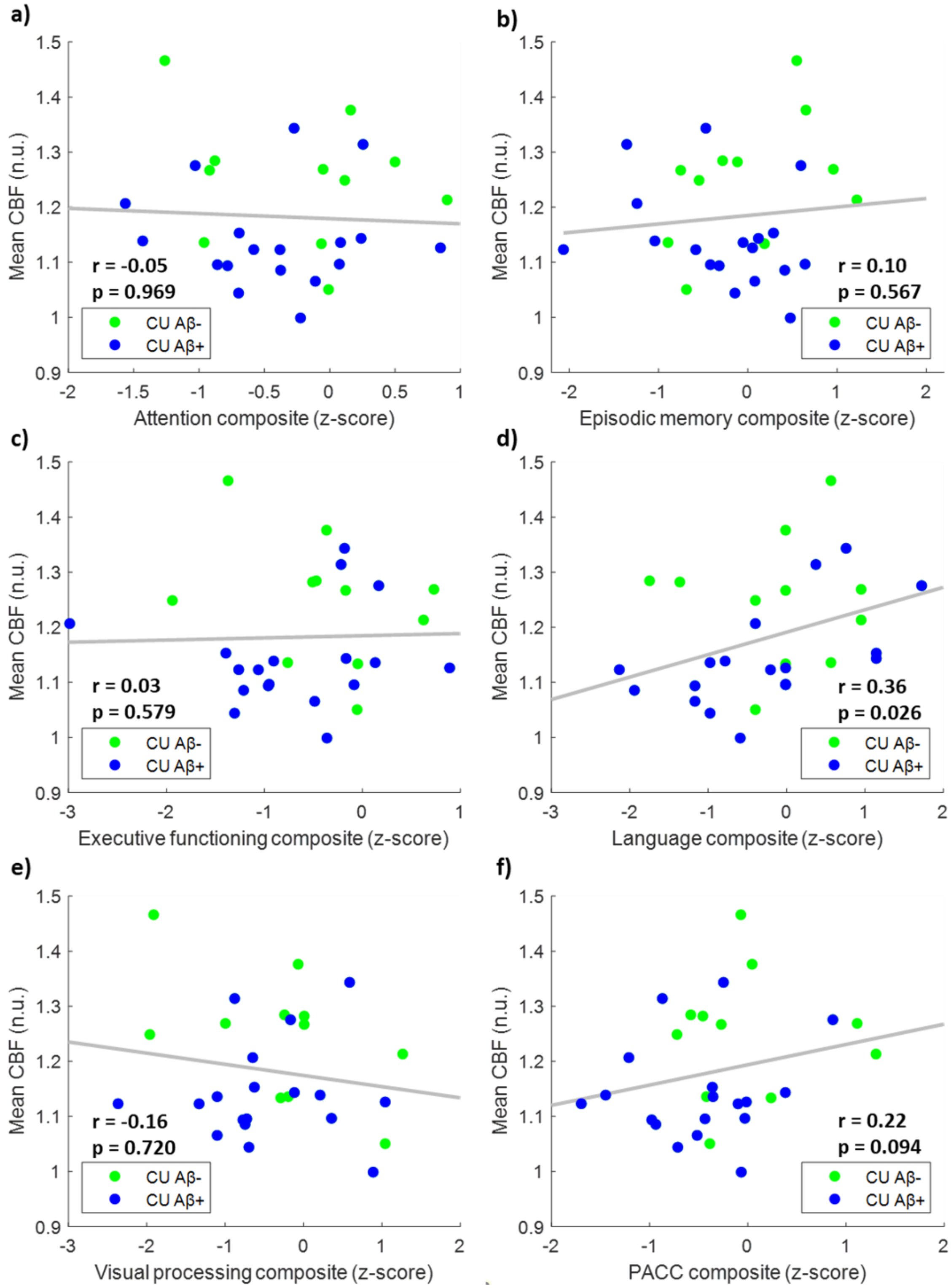

FIGURE S12: Association of CBF within a first data-driven mask with cognitive scores in CU individuals.

The first data-driven mask consisted of areas of the brain where te-ASL revealed significantly reduced CBF in CI Aβ+ individuals, in comparison with CU Aβ- individuals. Scatterplots representing the association of mean CBF with composite scores of: **a)** attention (11 CU Aβ- and 18 CU Aβ+ subjects); **b)** episodic memory (11 CU Aβ- and 18 CU Aβ+ subjects); **c)** executive functioning (11 CU Aβ- and 18 CU Aβ+ subjects); **d)** language (11 CU Aβ- and 18 CU Aβ+ subjects); **e)** visual processing (11 CU Aβ- and 18 CU Aβ+ subjects); and **f)** PACC (11 CU Aβ- and 18 CU Aβ+ subjects). FDR-corrected *p*-values are reported in the "Results" section. Mean CBF was not associated with the scores of any cognitive variables studied.

Aβ: amyloid-beta; Aβ-: normal levels of Aβ proteins; Aβ+: altered levels of Aβ proteins; ASL: arterial spin labeling; CBF: cerebral blood flow; CI: cognitively impaired; CU: cognitively unimpaired; n.u.: normalized units; PACC: Preclinical Alzheimer Cognitive Composite; te: time-encoded.

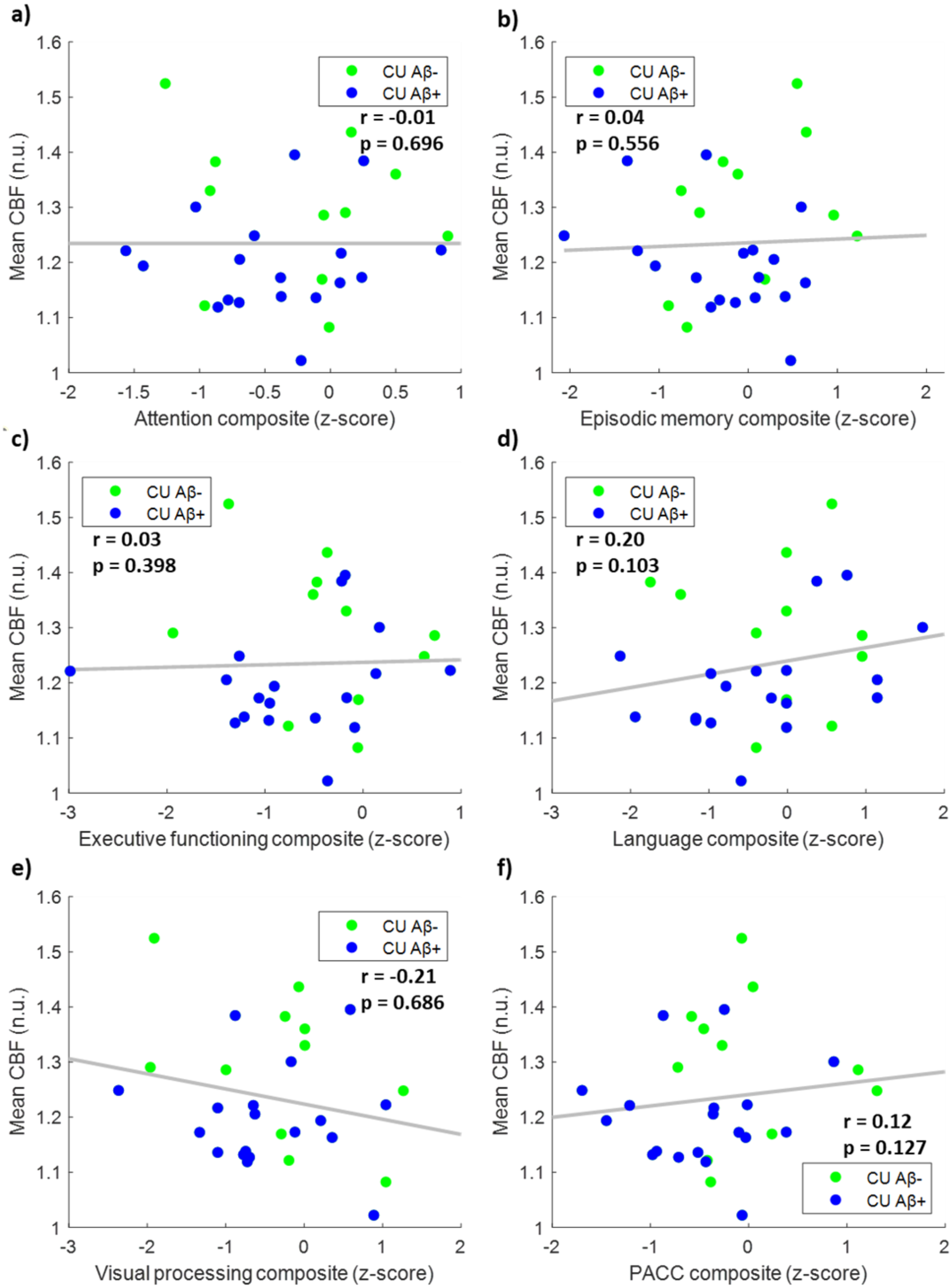

FIGURE S13: Association of CBF within an *a priori* mask with cognitive scores in CU individuals.

The *a priori* mask consisted of areas of the brain associated with CBF reduction in AD, as previously reported [11]. Scatterplots representing the association of mean CBF with composite scores of: **a)** attention (11 CU Aβ- and 18 CU Aβ+ subjects); **b)** episodic memory (11 CU Aβ- and 18 CU Aβ+ subjects); **c)** executive functioning (11 CU Aβ- and 18 CU Aβ+ subjects); **d)** language (11 CU Aβ- and 18 CU Aβ+ subjects); **e)** visual processing (11 CU Aβ- and 18 CU Aβ+ subjects); and **f)** PACC (11 CU Aβ- and 18 CU Aβ+ subjects). FDR-corrected *p*-values are reported in the "Results" section. Mean CBF was not associated with the scores of any cognitive variables studied.

Aβ: amyloid-beta; Aβ-: normal levels of Aβ proteins; Aβ+: altered levels of Aβ proteins; AD: Alzheimer's disease; CBF: cerebral blood flow; CU: cognitively unimpaired; n.u.: normalized units; PACC: Preclinical Alzheimer Cognitive Composite.

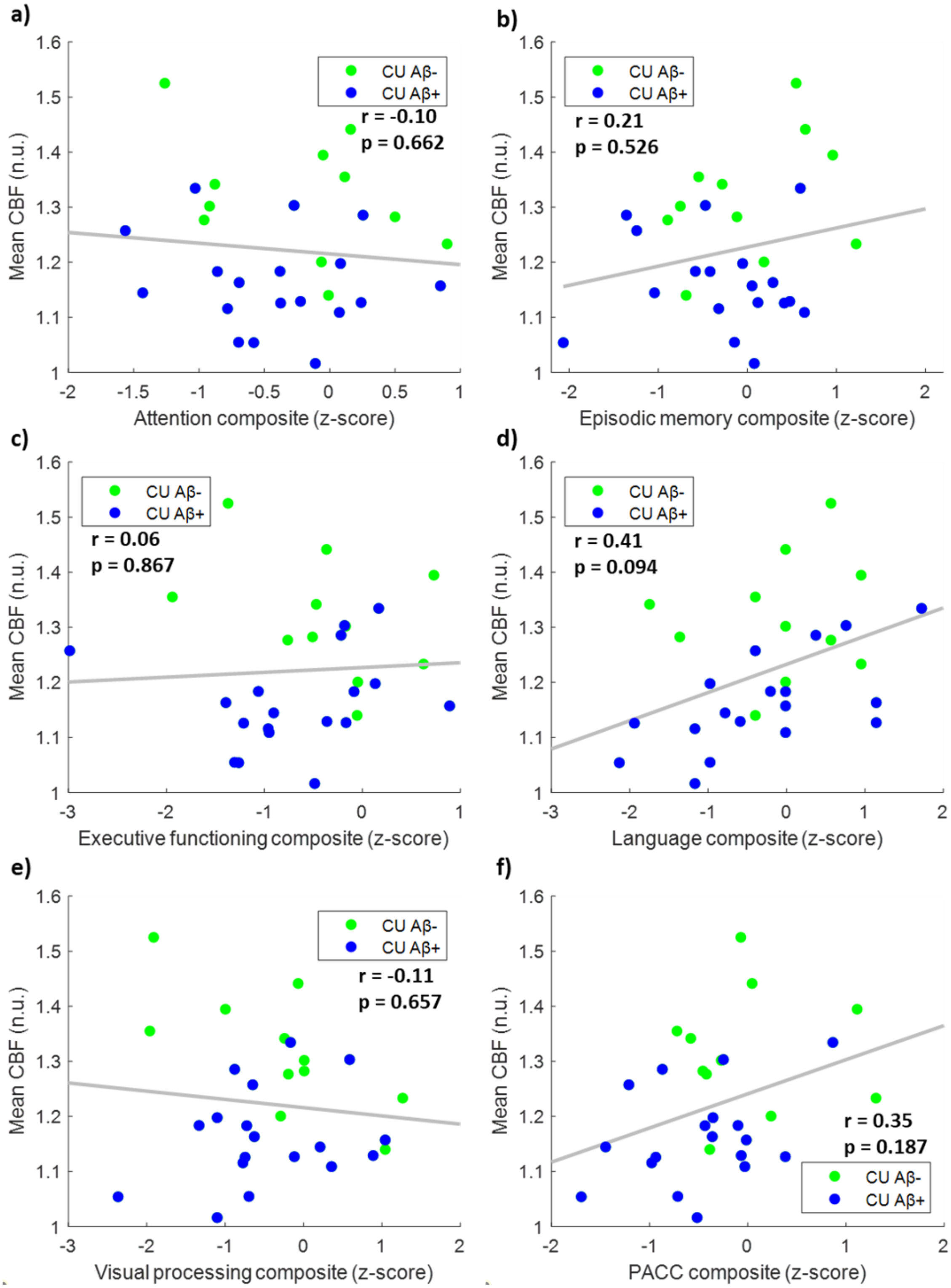

FIGURE S14: Association of CBF within a second data-driven mask with cognitive scores in CU individuals.

The second data-driven mask consisted of areas of the brain where te-ASL revealed significantly reduced CBF in CU Aβ+ individuals, in comparison with CU Aβ- individuals. Scatterplots representing the association of mean CBF with **a)** attention (11 CU Aβ- and 18 CU Aβ+ subjects); **b)** episodic memory (11 CU Aβ- and 18 CU Aβ+ subjects); **c)** executive functioning (11 CU Aβ- and 18 CU Aβ+ subjects); **d)** language (11 CU Aβ- and 18 CU Aβ+ subjects); **e)** visual processing (11 CU Aβ- and 18 CU Aβ+ subjects); and **f)** PACC. FDR-corrected *p*-values are reported in the "Results" section. Lower CBF was associated with a lower language composite score. Aβ: amyloid-beta; Aβ-: normal levels of Aβ proteins; Aβ+: altered levels of Aβ proteins; ASL: arterial spin labeling; CBF: cerebral blood flow; CU: cognitively unimpaired; n.u.: normalized units; PACC: Preclinical Alzheimer Cognitive Composite; te: time-encoded.

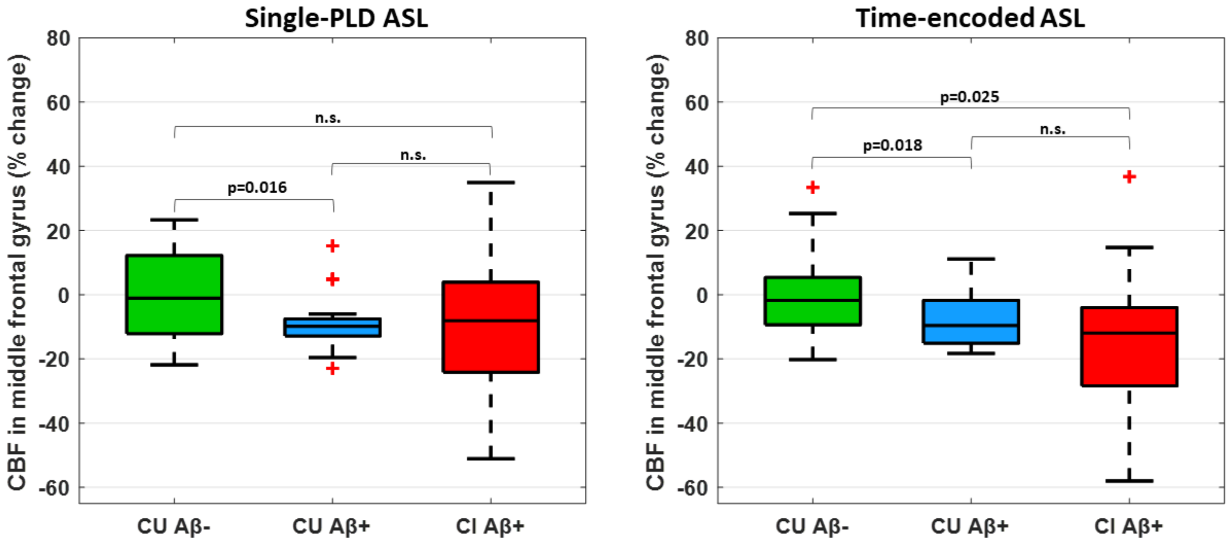

FIGURE S15. Percentage change in mean CBF in comparison with the CU Aβ- group computed within the middle frontal gyrus.

CBF averaged across voxels in the middle frontal cortex expressed in percentage change with respect to the CU Aβ- group. te-ASL analysis revealed reduced CBF in the middle frontal gyrus of CI Aβ+ individuals as compared to CU Aβ- individuals.

Aβ: amyloid-beta; Aβ-: normal levels of Aβ proteins; Aβ+: altered levels of Aβ proteins; AD: Alzheimer's disease; ASL: arterial spin labeling; CBF: cerebral blood flow; CI: cognitively impaired; CU: cognitively unimpaired; n.s.: non-significant; PLD: post-label delay; te: time-encoded.
